# Effects of Time-Restricted Eating on Nonalcoholic Fatty Liver Disease: The TREATY-FLD Randomized Clinical Trial

**DOI:** 10.1001/jamanetworkopen.2023.3513

**Published:** 2023-03-17

**Authors:** Xueyun Wei, Bingquan Lin, Yan Huang, Shunyu Yang, Chensihan Huang, Linna Shi, Deying Liu, Peizhen Zhang, Jiayang Lin, Bingyan Xu, Dan Guo, Changwei Li, Hua He, Shiqun Liu, Yaoming Xue, Yikai Xu, Huijie Zhang

**Affiliations:** 1 Department of Endocrinology and Metabolism, Nanfang Hospital, Southern Medical University, Guangzhou, China; 2 Imaging Center, Nanfang Hospital, Southern Medical University, Guangzhou, China; 3 Department of Food Safety and Health Research Center, School of Public Health, Southern Medical University, Guangzhou, China; 4 Department of Nutrition, Nanfang Hospital, Southern Medical University, Guangzhou, China; 5 Department of Epidemiology, Tulane University School of Public Health and Tropical Medicine, New Orleans, Louisiana

## Abstract

**Importance:**

The efficacy and safety of time-restricted eating (TRE) on nonalcoholic fatty liver disease (NAFLD) remain uncertain.

**Objective:**

To compare the effects of TRE vs daily calorie restriction (DCR) on intrahepatic triglyceride (IHTG) content and metabolic risk factors among patients with obesity and NAFLD.

**Design, Setting, and Participants:**

This 12-month randomized clinical trial including participants with obesity and NAFLD was conducted at the Nanfang Hospital in Guangzhou, China, between April 9, 2019, and August 28, 2021.

**Interventions:**

Participants with obesity and NAFLD were randomly assigned to TRE (eating only between 8:00 am and 4:00 pm) or DCR (habitual meal timing). All participants were instructed to maintain a diet of 1500 to 1800 kcal/d for men and 1200 to 1500 kcal/d for women for 12 months.

**Main Outcomes and Measures:**

The primary outcome was change in IHTG content measured by magnetic resonance imaging; secondary outcomes were changes in body weight, waist circumference, body fat, and metabolic risk factors. Intention-to-treat analysis was used.

**Results:**

A total of 88 eligible patients with obesity and NAFLD (mean [SD] age, 32.0 [9.5] years; 49 men [56%]; and mean [SD] body mass index, 32.2 [3.3]) were randomly assigned to the TRE (n = 45) or DCR (n = 43) group. The IHTG content was reduced by 8.3% (95% CI, −10.0% to −6.6%) in the TRE group and 8.1% (95% CI, −9.8% to −6.4%) in the DCR group at the 6-month assessment. The IHTG content was reduced by 6.9% (95% CI, −8.8% to −5.1%) in the TRE group and 7.9% (95% CI, −9.7% to −6.2%) in the DCR group at the 12-month assessment. Changes in IHTG content were comparable between the 2 groups at 6 months (percentage point difference: −0.2; 95% CI, −2.7 to 2.2; *P* = .86) and 12 months (percentage point difference: 1.0; 95% CI, −1.6 to 3.5; *P* = .45). In addition, liver stiffness, body weight, and metabolic risk factors were significantly and comparably reduced in both groups.

**Conclusions and Relevance:**

Among adults with obesity and NAFLD, TRE did not produce additional benefits for reducing IHTG content, body fat, and metabolic risk factors compared with DCR. These findings support the importance of caloric intake restriction when adhering to a regimen of TRE for the management of NAFLD.

**Trial Registration:**

ClinicalTrials.gov Identifiers: NCT03786523 and NCT04988230

## Key Points

**Question:**

Is time-restricted eating more effective in improving nonalcoholic fatty liver disease than daily calorie restriction?

**Findings:**

In this randomized clinical trial including 88 patients with obesity and nonalcoholic fatty liver disease, the intrahepatic triglyceride content was reduced by 6.9% in the time-restricted eating group and 7.9% in the daily calorie restriction group during 12 months, but with no significant between-group differences. Time-restricted eating also did not produce additional benefits for reducing body fat or major metabolic risk factors compared with daily calorie restriction.

**Meaning:**

The findings of this randomized clinical trial support the importance of caloric restriction with use of time-restricted eating among adults with obesity and nonalcoholic fatty liver disease.

## Teaser

This randomized clinical trial compares the use of time-restricted eating with daily calorie restriction in adults with obesity and nonalcoholic fatty liver disease.

## Introduction

Nonalcoholic fatty liver disease (NAFLD) has become a major worldwide public health challenge.^1^ It affects approximately 20% to 30% of adults in the general population, and more than 70% of patients with obesity and diabetes have NAFLD.^2,3,4,5^ Approximately 29.2% of adults in the general population have NAFLD in China.^6^ It is closely related to obesity, type 2 diabetes, hyperlipidemia, and hypertension and has been associated with an increased risk of cardiovascular diseases.^1,7^ Weight loss via lifestyle modifications has been documented to improve liver fat and metabolic disorders.^8^

Dietary calorie restriction has been proven to be effective in reducing weight and intrahepatic lipid levels among patients with NAFLD.^9,10,11^ Nevertheless, long-term adherence to lifestyle modification is difficult. Time-restricted eating (TRE) is one of the most popular intermittent fasting regimens involving a specific eating period within a 24-hour cycle. The TRE regimen has gained attention because it reduces weight and enhances adherence.^12,13^ Studies in rodents suggest that food timing rather than calorie intake underlies the beneficial effects of TRE regimen.^14,15^ Evidence indicates that fat storage increases during the day and is the greatest after an evening meal.^16^ Observational studies suggest that eating meals later in the day may be associated with the success of weight loss therapy in humans.^17,18^ Several pilot clinical trials reported that TRE can result in reduced calorie intake and is associated with a decrease in body weight and fat mass in individuals with obesity.^19,20,21,22^ However, most of the reported benefits of TRE are either untested or undertested in humans and cannot isolate the effects of TRE itself. A small clinical trial reported that the regimen of eating 2 meals (eating periods from 6:00 am to 4:00 pm) reduced intrahepatic lipids measured by proton magnetic resonance spectroscopy compared with the control regimen (eating 6 smaller meals) among 54 patients with type 2 diabetes during 12 weeks’ intervention.^23^ To date, the efficacy of TRE on NAFLD is uncertain. Furthermore, to our knowledge, no studies compared the effects of TRE and daily calorie restriction (DCR) on intrahepatic lipid levels in patients with NAFLD.

The Time Restricted Feeding on Nonalcoholic Fatty Liver Disease (TREATY-FLD) randomized clinical trial aimed to compare the effects of TRE vs DCR on intrahepatic triglyceride (IHTG) content and metabolic risk factors among patients with obesity and NAFLD. We hypothesized that 8-hour TRE would be more effective than DCR in improving NAFLD and metabolic risk factors.

## Methods

### Study Design

This randomized, parallel-group, observer-blinded clinical trial was designed to compare the effects of 8-hour TRE vs DCR on the IHTG content and metabolic risk factors among patients with NAFLD. Eligible trial participants were randomly assigned to the TRE or DCR program for 12 months. The duration of intervention of the original study design was 6 months (registered as NCT03786523); at the beginning of the study, we revised the design and prolonged the intervention to 12 months to compare the long-term effects of TRE vs DCR on NAFLD (registered separately as NCT04988230). The duration of the intervention included the original designed 6 months and the next 6 months follow-up visits. The trial protocol and statistical analysis plan are available in Supplement 1. Patient recruitment and intervention were conducted from April 9, 2019, through August 28, 2021, at the Nanfang Hospital in Guangzhou, China. The trial was overseen by a steering committee affiliated with the Southern Medical University Institutional Review Board. The study protocol and informed consent form were approved by institutional review boards of the Nanfang Hospital of Southern Medical University. All patients provided written informed consent before enrollment; no financial compensation was provided. The study follows the Consolidated Standards of Reporting Trials (CONSORT) reporting guideline for randomized clinical trials.

### Participants

All study participants were recruited from the public via promotional leaflets, posters, internet, and community screenings. All interested persons were prescreened to identify potential individuals aged 18 to 75 years with obesity (body mass index [BMI] between 28.0 and 45.0 [calculated as weight in kilograms divided by height in meters squared]) and ultrasonography-diagnosed NAFLD. After the prescreening, potential participants were invited to attend a screening magnetic resonance imaging examination at the study clinic. Those who had NAFLD confirmed by magnetic resonance imaging (IHTG content ≥5%) were enrolled in this study. Among the criteria for exclusion were acute or chronic viral hepatitis, drug-induced liver disease, autoimmune hepatitis, diabetes, serious liver dysfunction, chronic kidney disease, excessive alcohol consumption (>20 g/d for women or >30 g/d for men), serious cardiovascular or cerebrovascular disease within 6 months, severe gastrointestinal diseases or gastrointestinal surgery in the past 12 months, active participation in a weight loss program, use of medications that affect weight or energy balance, and current or planned pregnancy.

### Randomization and Blinding

Eligible participants were randomly assigned to the TRE or DCR group with an allocation ratio of 1:1. Randomization was conducted in a block size of 6. The computer-generated randomization sequence was prepared by an independent researcher who was not involved in the study. Investigators who assessed the study outcomes and analyzed the data were blinded to the group assignment.

### Intervention Programs

All participants were instructed to follow a diet of 1500 to 1800 kcal/d for men and 1200 to 1500 kcal/d for women. The diets were composed of 40% to 55% carbohydrate, 15% to 20% protein, and 20% to 30% fat.^24^ All participants were provided with 1 protein shake (Nutriease; Zhejiang Nutriease Co) per day for the first 6 months and received dietary counseling for the duration of the study. Participants assigned to the TRE group were instructed to consume the prescribed calories from 8:00 am to 4:00 pm every day, and only noncaloric beverages were permitted outside of the daily eating window. Participants in the DCR group had no eating time restriction during the 12-month study period.

Dietary counseling was conducted by trained nutritionists. Participants received written dietary information booklets, which had food portion advice and sample menus of similar dietary energy restrictions in accordance with the Dietary Guidelines for macronutrient intake.^24,25^ Participants were encouraged to weigh foods to ensure accuracy of intake. All participants were required to write a dietary log and record daily food pictures and mealtimes on a custom mobile study application. All participants received follow-up telephone calls or a text message through the study app about their energy intake twice per week. The trained nutritionists also met with study participants individually every 2 weeks to assess their adherence to the program and provide suggestions for improvements and personalized energy targets during the first 6 months of the trial. Participants were instructed to maintain their diet regimens during the next 6-month follow-up visit and write in their dietary log and record food pictures and mealtimes 3 times per week. In this phase, participants received follow-up telephone calls or a text message through the study app once per week and met with the nutritionist monthly. Dietary intake and mealtimes were assessed daily using each participant’s log and timely recorded food photographs based on the nutrient content listed in the China Food Composition Tables.^26^ All participants attended health education sessions monthly over 12 months and were instructed not to change their physical activity habits throughout the trial.

### Adherence to the Intervention Programs

Adherence to the diet program was evaluated as days that participants met the requirements of the diet program. In the TRE group, participants were required to both eat within the prescribed eating period and meet the daily caloric intake goal. In the DCR group, participants were required to consume the prescribed daily energy amount.

### Outcomes

The primary outcome was change in the IHTG content from baseline to 6 and 12 months. The IHTG content was measured using magnetic resonance imaging (Ingenia 3.0T mDIXON Quant; Philips Healthcare)^27,28,29^ at baseline, 6 months, and 12 months. The secondary outcomes were changes in body weight, BMI, waist circumference, body fat mass, lean mass, liver stiffness, liver enzyme levels, and other metabolic risk factors, including plasma glucose levels, serum lipid levels, and blood pressure. Body fat mass and lean mass were quantified using a whole-body dual x-ray system (Lunar iDXA; GE Healthcare). Abdominal visceral fat and subcutaneous fat areas were measured by computed tomography (Revolution; GE Healthcare) at the level of the lumbar vertebrae.^30^ Liver stiffness was assessed by transient elastography (FibroScan 502 Touch; Echosens). Metabolic risk factors and liver enzyme levels were measured using standard methods at baseline and the 6- and 12-month follow-up visits.

Nutrient intake was estimated by 3 consecutive 24-hour dietary recalls (2 weekdays and 1 weekend day) at baseline and 6 months. Nutrient intake was calculated based on the China Food Composition Tables. Physical activity was assessed using the International Physical Activity Questionnaire at baseline, 6 months, and 12 months.^31^ Additional outcomes included quality of life as measured according to the 12-item Short-Form Health Survey Questionnaire (SF-12),^32^ depressive symptoms as measured by the Patient Health Questionnaire-9,^33^ and sleep quality as measured by the Pittsburgh Sleep Quality Index.^34^

### Statistical Analysis

We estimated that with a sample size of 68 individuals, the trial would provide greater than 90% statistical power to detect a significant difference of 0.8% (unit value) in the reduction of IHTG content (SD, 1.0%) between the TRE group and the DCR group at a significance level of .05 using a 2-tailed test. The expected group difference and SD of reduction in IHTG content were based on preliminary data for comparison between the TRE regimen with caloric intake restriction and regular caloric intake (no time restriction).^23,35^ Accounting for an 80% follow-up rate, a total of 88 participants were enrolled in this trial.

Data were analyzed according to participants’ randomization assignment (intention-to-treat). PROC MIXED of SAS statistical software, version 9.4 (SAS Institute Inc) was used to obtain point estimates and SEs of the treatment effects and to test for differences between treatments. Group differences in the study outcomes were evaluated using the general linear model for continuous variables and the χ^2^ test for categorical variables. We also used a linear mixed-effects model to compare the effects of the 2 diet programs on the IHTG content and main outcomes. In the linear mixed model, an autoregressive correlation matrix was used to correct within-participant correlation for repeated measurements, participants were treated as a random effect, and intervention group, follow-up time, and their 2-factor interactions were assumed to be estimable fixed effects. Missing data were handled by multiple imputations (n = 20) at random using the Markov chain Monte Carlo method. Data are presented as least-squares means with 95% CIs for continuous variables and risk ratios for categorical outcomes. *P* < .05 was considered statistically significant.

## Results

**Figure 1.**
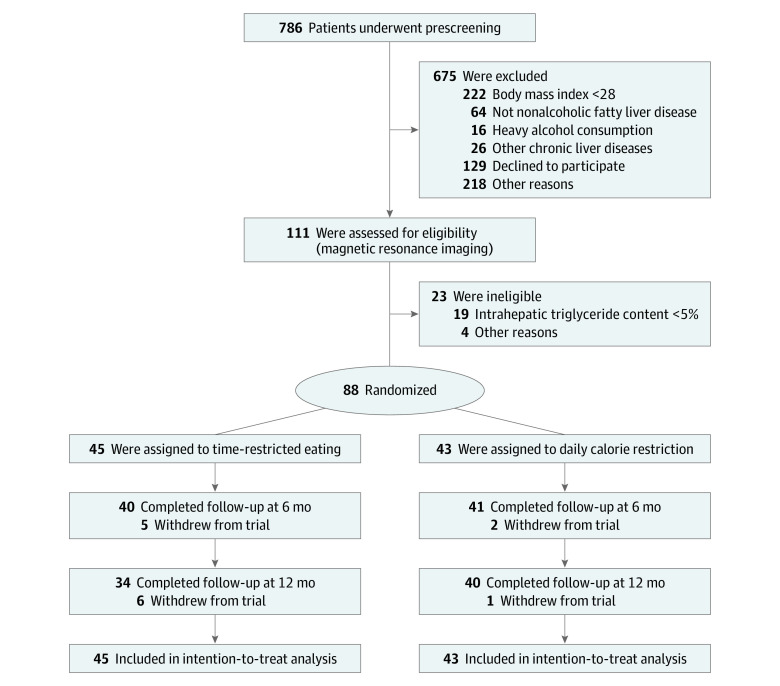
Flowchart of Trial Participants

A total of 88 eligible patients with obesity and NAFLD (mean [SD] age, 32.0 [9.5] years; 49 men [56%]; 39 women [44%]; and mean [SD] BMI, 32.2 [3.3]) were randomly assigned to the TRE (n = 45) or DCR (n = 43) group (Figure 1). Of those participants, 81 (92%) completed the 6-month intervention and 74 (84%) completed the entire 12-month intervention. Baseline characteristics had comparable distribution between the TRE and DCR groups (Table 1).

**Table 1.** Baseline Characteristics of Study Participants

Variable^a^	Mean (SD)	
	TRE (n = 45)	DCR (n = 43)
Sex, No. (%)		
Female	21 (47)	18 (42)
Male	24 (53)	25 (58)
Age, y	32.3 (10.5)	31.7 (8.3)
High school education, No. (%)	44 (98)	42 (98)
Weight, kg	88.9 (10.9)	91.5 (13.6)
BMI	32.2 (3.4)	32.2 (3.2)
Waist circumference, cm	100.4 (8.2)	102.3 (9.5)
Body fat percent, %	38.3 (6.1)	38.7 (5.2)
Fat mass, kg	33.8 (7.4)	35.0 (7.2)
Lean mass, kg	51.4 (7.9)	52.7 (8.8)
Fat area, median (IQR), cm^2^		
Total abdominal	466.9 (391.0-511.2)	452.0 (404.4-565.2)
Subcutaneous	325.3 (277.8-384.4)	327.4 (260.0-386.1)
Visceral	129.9 (101.3-171.1)	144.2 (110.1-174.4)
Visceral to subcutaneous fat ratio, median (IQR), %	39.5 (27.1-58.7)	41.5 (30.8-60.0)
Intrahepatic triglyceride content, median (IQR), %	13.1 (8.7-23.3)	13.8 (9.2-20.3)
Liver stiffness, median (IQR), kPa	6.5 (5.0-8.8)	6.8 (5.4-8.1)
Blood pressure, mm Hg		
Systolic	125.8 (11.8)	128.2 (12.1)
Diastolic	73.9 (9.3)	77.4 (9.8)
Pulse, beats/min	81.0 (12.4)	80.3 (9.5)
Triglycerides, median (IQR), mg/dL	155.9 (106.3-183.4)	149.7 (122.3-202.9)
Total cholesterol, mg/dL	194.6 (34.6)	201.9 (40.7)
HDL-C, mg/dL	42.9 (9.0)	43.3 (10.3)
LDL-C, mg/dL	131.3 (30.8)	132.7 (35.9)
Plasma glucose, mg/dL	92.6 (12.2)	93.4 (18.9)
Hemoglobin A_1c_, %	5.4 (0.5)	5.5 (0.7)
HOMA-IR, median (IQR)	3.7 (2.7-6.0)	3.4 (2.2-4.4)
Intake		
Energy, kcal/d	2111.7 (303.0)	2161.4 (333.4)
Fat, %	35.7 (5.9)	35.1 (5.4)
Protein, %	16.8 (2.8)	16.8 (3.0)
Carbohydrate, %	48.0 (6.9)	48.3 (7.2)
Alcohol, median (IQR), g/wk	0.0 (0.0-18.7)	0.0 (0.0-11.7)
Physical activity, median (IQR), MET/wk	10.7 (7.7-21.2)	14.6 (6.6-23.1)
SF-12 score		
Physical component summary	44.5 (7.0)	44.9 (6.5)
Mental component summary	54.0 (7.0)	54.4 (7.4)
Total PSQI sleep quality score	5.3 (2.4)	6.1 (2.8)
Total PHQ-9 score, median (IQR)	3 (1-5)	3 (2-6)

Abbreviations: BMI, body mass index (calculated as weight in kilograms divided by height in meters squared); DCR, daily calorie restriction; HDL-C, high-density lipoprotein cholesterol; HOMA-IR, homeostasis model assessment of insulin resistance (calculated as insulin × glucose/405, where the unit of measure for insulin is in microinternational units per milliliter and the unit of measure for glucose is milligrams per deciliter); LDL-C, low-density lipoprotein cholesterol; MET, metabolic equivalent; PHQ-9, Patient Health Questionnaire-9; PSQI, Pittsburgh Sleep Quality Index; SF-12, 12-item Short-Form Health Survey Questionnaire; TRE, time-restricted eating.

SI conversion factors: To convert glucose to millimoles per liter, multiply by 0.0555; cholesterol (HDL-C, LDL-C, and total) to millimoles per liter, multiply by 0.0259; hemoglobin A_1c_ to proportion of total hemoglobin, multiply by 0.01; and triglycerides to millimoles per liter, multiply by 0.0113.

^a^ No. (%) of missing values. At baseline: fat mass, 1 (1%); lean mass, 1 (1%). At 6 months: weight, 7 (8%); BMI, 7 (8%); waist circumference, 7 (8%); systolic blood pressure, 7 (8%); diastolic blood pressure, 7 (8%); fat mass, 7 (8%); lean mass, 7 (8%); visceral fat area, 7 (8%); subcutaneous fat area, 7 (8%); plasma glucose, 7 (8%); total cholesterol, 7 (8%); triglycerides, 7 (8%); LDL-C, 7 (8%); and HDL-C, 7 (8%). At 12 months: weight, 14 (16%); BMI, 14 (16%); waist circumference, 15 (17%); systolic blood pressure, 15 (17%); diastolic blood pressure, 15 (17%); fat mass, 18 (20%); lean mass, 18 (20%); visceral fat area, 15 (17%); subcutaneous fat area, 15 (17%); plasma glucose, 15 (17%); total cholesterol, 15 (17%); triglycerides, 15 (17%); LDL-C, 15 (17%); and HDL-C, 15 (17%).

The mean (SD) percentage of days that participants adhered to both the prescribed calories and eating period was 85.0% (10.7%) in the TRE group and 85.7% (9.4%) in the DCR group during 12 months (eTable 1 in Supplement 2). The average daily energy deficit and percentage of energy intake from carbohydrates, fat, and protein were similar in the 2 groups during 12 months. By design, the mean daily eating duration in the TRE group was significantly shorter than that of the DCR group. Physical activity was also similar between the 2 diet groups and was stable during 12 months. Scores on the SF-12 physical and mental components, Patient Health Questionnaire-9 depression module, and Pittsburgh Sleep Quality Index were similar in the 2 groups.

### Primary Outcome

**Figure 2.**
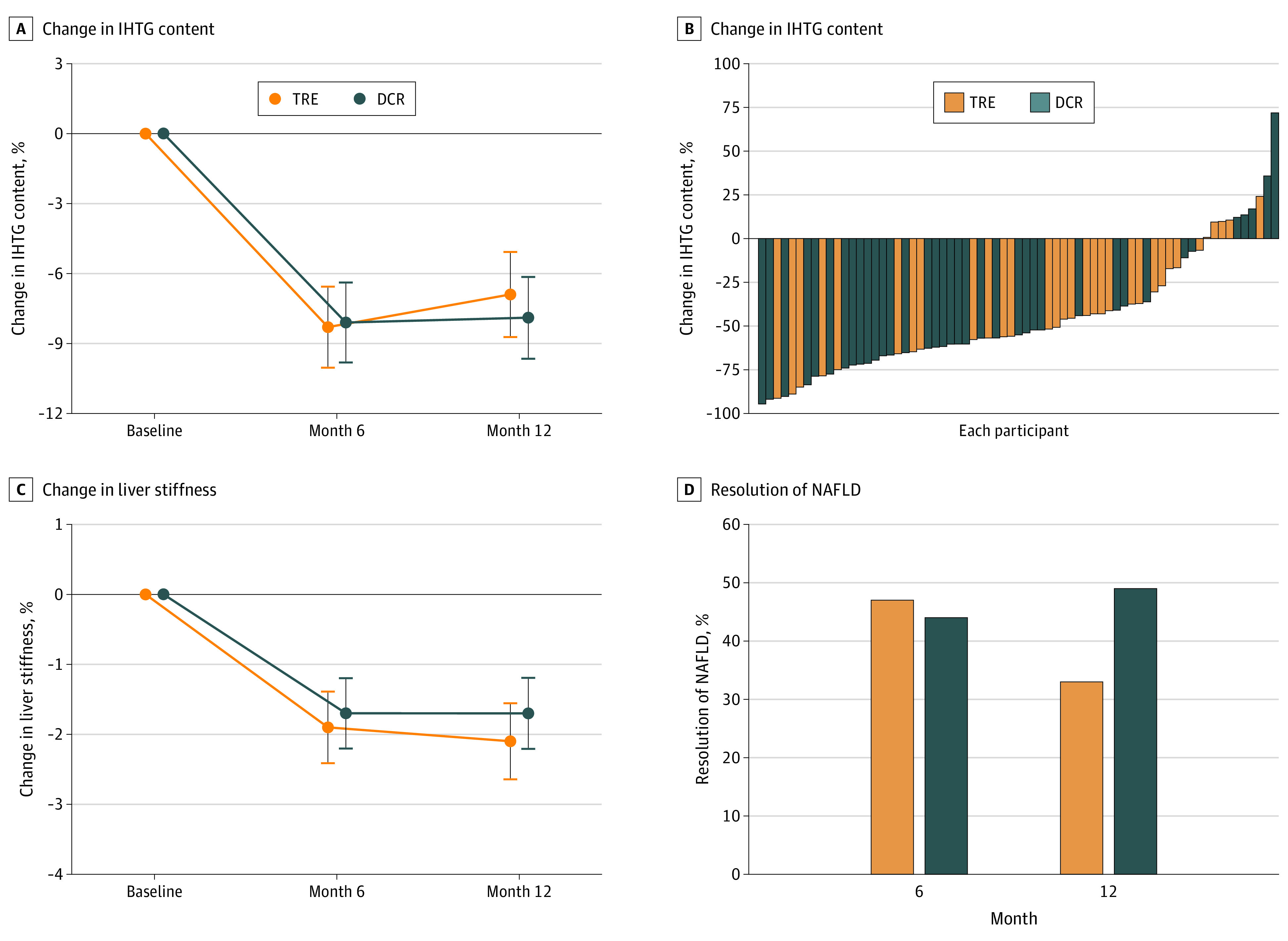
Effect of Time-Restricted Eating (TRE) vs Daily Calorie Restriction (DCR) on the Intrahepatic Triglyceride (IHTG) Content A, Change in IHTG content. Data are presented as estimated absolute change of IHTG content. Error bars represent 95% CIs. B, Percentage of IHTG content change for each participant. C, Change in liver stiffness. Data are presented as estimated absolute change of liver stiffness. Error bars represent 95% CIs. D, Percentage of patients with resolution of nonalcoholic fatty liver disease (NAFLD) at 6-month (*P* = .40) and 12-month (*P* = .31) assessment. Resolution of NAFLD is defined as IHTG content less than 5%.

The IHTG content was reduced by 8.3% (95% CI, −10.0% to −6.6%) at 6 months and 6.9% (95% CI, −8.8% to −5.1%) at 12 months in the TRE group. Likewise, it was reduced by 8.1% (95% CI, −9.8% to −6.4%) at the 6-month assessment and 7.9% (95% CI, −9.7% to −6.2%) at 12 months in the DCR group. However, the net change in IHTG content was not significantly different between the groups at the 6-month (percentage point difference: −0.2; 95% CI, −2.7 to 2.2; *P* = .86) or 12-month (percentage point difference: 1.0; 95% CI, −1.6 to 3.5; *P* = .45) assessments (Figure 2). Liver stiffness was reduced by 2.1 kPa (95% CI, −2.7 to −1.6 kPa) in the TRE group and 1.7 kPa (95% CI, −2.3 to −1.2 kPa) in the DCR group at 12 months, with no significant difference between the 2 groups (*P* = .33). The percentages of participants with resolution of NAFLD (defined as IHTG content <5%) at month 12 were similar in the 2 groups (TRE group, 33% vs DCR group, 49%; *P* = .31). Sensitivity analysis using multiple imputed data showed similar results for the primary outcomes (eTable 2 in Supplement 2). Furthermore, the IHTG content reductions were similar for the 2 regimens when assessed according to adherence to the prescribed diet (eFigure 1 in Supplement 2).

### Weight Loss and Body Fat

During the 12-month intervention, body weight was significantly reduced by 8.4 kg (95% CI, −10.3 to −6.4 kg) in the TRE group and 7.8 kg (95% CI, −9.7 to −5.9 kg) in the DCR group, with no significant between-group differences (−0.6 kg; 95% CI, −3.3 to 2.2 kg; *P* = .69) (Table 2; eFigure 2 in Supplement 2). Likewise, waist circumference, body fat percentage, fat mass, lean mass, total abdominal fat, subcutaneous fat, visceral fat, and visceral to subcutaneous fat ratio were all significantly reduced in the 2 groups, with no significant between-group differences.

**Table 2.** Effects of Diets on Weight Loss and Body Composition

Outcome	Change (95% CI)		Difference between groups (95% CI)	*P* value^a^
	TRE (n = 45)	DCR (n = 43)		
**Weight, kg**				
Month 6	−9.8 (−11.7 to −7.9)	−9.7 (−11.6 to −7.9)	−0.1 (−2.8 to 2.6)	.94
Month 12	−8.4 (−10.3 to −6.4)	−7.8 (−9.7 to −5.9)	−0.6 (−3.3 to 2.2)	.69
**BMI**				
Month 6	−3.6 (−4.3 to −2.9)	−3.4 (−4.1 to −2.8)	−0.2 (−1.1 to 0.8)	.70
Month 12	−3.1 (−3.8 to −2.4)	−2.8 (−3.5 to −2.1)	−0.3 (−1.3 to 0.6)	.51
**Waist circumference, cm**				
Month 6	−10.0 (−12.1 to −8.0)	−9.1 (−11.1 to −7.1)	−0.9 (−3.8 to 2.0)	.53
Month 12	−9.3 (−11.4 to −7.2)	−8.2 (−10.2 to −6.2)	−1.1 (−4.0 to 1.9)	.47
**Body fat percentage, %**				
Month 6	−4.8 (−6.1 to −3.6)	−4.6 (−5.8 to −3.3)	−0.3 (−2.0 to 1.5)	.77
Month 12	−4.6 (−5.9 to −3.3)	−3.7 (−5.0 to −2.5)	−0.9 (−2.7 to 0.9)	.34
**Fat mass, kg**				
Month 6	−7.1 (−8.5 to −5.6)	−7.0 (−8.4 to −5.6)	−0.1 (−2.1 to 2.0)	.96
Month 12	−6.1 (−7.6 to −4.6)	−5.8 (−7.2 to −4.4)	−0.3 (−2.4 to 1.8)	.77
**Lean mass, kg**				
Month 6	−2.3 (−3.0 to −1.7)	−2.1 (−2.7 to −1.5)	−0.2 (−1.1 to 0.7)	.61
Month 12	−2.1 (−2.8 to −1.4)	−1.8 (−2.4 to −1.2)	−0.3 (−1.2 to 0.6)	.54
**Total abdominal fat, cm^2^**				
Month 6	−118.5 (−146.7 to 90.3)	−101.9 (−129.7 to −74.1)	−16.6 (−56.2 to 23.0)	.41
Month 12	−94.0 (−123.5 to 64.6)	−86.6 (−114.5 to 58.7)	−7.4 (−48.0 to 33.2)	.72
**Subcutaneous fat, cm^2^**				
Month 6	−78.9 (−97.9 to −59.9)	−59.4 (−78.2 to −40.7)	−19.5 (−46.2 to 7.2)	.15
Month 12	−60.0 (−79.9 to −40.2)	−51.8 (−70.6 to −33.0)	−8.2 (−35.6 to 19.1)	.55
**Visceral fat, cm^2^**				
Month 6	−41.6 (−52.8 to −30.4)	−38.6 (−49.6 to −27.6)	−2.9 (−18.7 to 12.8)	.71
Month 12	−36.6 (−48.5 to −24.8)	−33.6 (−44.7 to −22.5)	−3.0 (−19.3 to 13.3)	.71
**Visceral to subcutaneous fat ratio, %**				
Month 6	−2.8 (−5.7 to 0.1)	−4.4 (−7.3 to −1.6)	1.7 (−2.4 to 5.8)	.42
Month 12	−4.9 (−8.1 to −1.7)	−4.7 (−7.6 to −1.8)	−0.2 (−4.5 to 4.1)	.92
**Liver stiffness, kPa**				
Month 6	−1.9 (−2.5 to −1.4)	−1.7 (−2.2 to −1.2)	−0.2 (−0.9 to 0.5)	.57
Month 12	−2.1 (−2.7 to −1.6)	−1.7 (−2.3 to −1.2)	−0.4 (−1.1 to 0.4)	.33

Abbreviations: BMI, body mass index (calculated as weight in kilograms divided by height in meters squared); DCR, daily calorie restriction; TRE, time-restricted eating.

^a^ For the between-group difference from linear mixed models that included diet, time, and diet × time interaction terms.

### Metabolic Risk Factors and Liver Enzymes

Metabolic risk factors, including systolic and diastolic blood pressure, pulse rate, and total cholesterol, triglyceride, high-density lipoprotein cholesterol, and low-density lipoprotein cholesterol levels were all significantly improved in the 2 groups over 12 months, with no significant between-group differences (Table 3). Both diets significantly reduced fasting plasma glucose level, hemoglobin A_1c_, and homeostasis model assessment of insulin resistance (HOMA-IR) at 6 months, and TRE significantly reduced HOMA-IR compared with DCR at 12 months. Similarly, both diets significantly reduced levels of liver enzymes, including serum alanine aminotransferase, aspartate aminotransferase, and γ-glutamyltransferase, with no significant between-group differences.

**Table 3.** Effects of Diets on Cardiovascular Risk Factors and Liver Enzymes

Outcome	Change (95% CI)		Difference between groups (95% CI)	*P* value^a^
	TRE (n = 45)	DCR (n = 43)		
**Systolic blood pressure, mm Hg**				
Month 6	−12.1 (−14.8 to −9.4)	−9.3 (−12.0 to −6.7)	−2.8 (−6.6 to 1.0)	.15
Month 12	−11.0 (−13.9 to −8.1)	−8.5 (−11.2 to −5.9)	−2.5 (−6.4 to 1.4)	.21
**Diastolic blood pressure, mm Hg**				
Month 6	−7.4 (−9.5 to −5.2)	−5.9 (−8.1 to −3.8)	−1.4 (−4.5 to 1.7)	.36
Month 12	−7.4 (−9.7 to −5.1)	−5.5 (−7.6 to −3.3)	−1.9 (−5.1 to 1.3)	.23
**Pulse, beats/min**				
Month 6	−4.9 (−7.7 to −2.1)	−3.4 (−6.2 to −0.6)	−1.4 (−5.4 to 2.5)	.47
Month 12	−5.3 (−8.3 to −2.3)	−3.1 (−5.9 to −0.3)	−2.2 (−6.3 to 1.9)	.29
**Triglycerides, mg/dL**				
Month 6	−62.4 (−78.7 to −46.0)	−55.3 (−71.5 to −39.1)	−7.1 (−30.1 to 15.9)	.54
Month 12	−39.0 (−56.7 to −21.2)	−38.0 (−54.3 to −21.6)	−1.0 (−25.1 to 23.1)	.93
**Total cholesterol, mg/dL**				
Month 6	−12.5 (−20.5 to −4.6)	−12.4 (−20.2 to −4.6)	−0.2 (−11.3 to 11.0)	.98
Month 12	−10.2 (−18.5 to −1.9)	−7.9 (−15.8 to −0.0)	−2.3 (−13.8 to 9.2)	.69
**HDL-C, mg/dL**				
Month 6	5.1 (2.9 to 7.3)	3.9 (1.7 to 6.0)	1.2 (−1.8 to 4.3)	.42
Month 12	6.5 (4.2 to 8.9)	3.7 (1.6 to 5.9)	2.8 (−0.4 to 6.0)	.08
**LDL-C, mg/dL**				
Month 6	−8.4 (−15.5 to −1.3)	−8.2 (−15.2 to −1.2)	−0.2 (−10.2 to −9.8)	.97
Month 12	−12.5 (−20.0 to −5.0)	−8.2 (−15.2 to −1.1)	−4.3 (−14.6 to 6.0)	.41
**Plasma glucose, mg/dL**				
Month 6	−6.2 (−11.2 to −1.2)	−5.1 (−10.0 to −0.2)	−1.1 (−8.1 to 5.9)	.76
Month 12	−5.9 (−11.3 to −0.5)	−0.8 (−5.8 to 4.2)	−5.1 (−12.4 to 2.3)	.17
**HOMA-IR**				
Month 6	−1.7 (−2.6 to −0.9)	−1.3 (−2.2 to −0.5)	−0.4 (−1.6 to 0.8)	.50
Month 12	−1.6 (−2.5 to −0.6)	−0.0 (−0.9 to 0.8)	−1.6 (−2.8 to −0.3)	.18
**Hemoglobin A_1c_, %**				
Month 6	−0.2 (−0.3 to −0.1)	−0.2 (−0.3 to −0.1)	0.0 (−0.1 to 0.2)	.59
Month 12	−0.2 (−0.3 to −0.1)	−0.1 (−0.2 to 0.0)	−0.1 (−0.3 to 0.1)	.38
**Alanine aminotransferase, U/L**				
Month 6	−14.4 (−20.3 to −8.5)	−17.1 (−22.9 to −11.3)	2.7 (−5.6 to 11.1)	.52
Month 12	−14.2 (−20.6 to −7.7)	−11.6 (−17.5 to −5.7)	−2.6 (−11.4 to 6.2)	.56
**Aspartate aminotransferase, U/L**				
Month 6	−5.3 (−8.6 to −2.0)	−7.2 (−10.4 to −4.0)	1.9 (−2.6 to 6.5)	.40
Month 12	−5.4 (−9.0 to −1.8)	−6.1 (−9.4 to −2.9)	0.7 (−4.1 to 5.6)	.76
**γ-Glutamyltransferase, U/L**				
Month 6	−11.7 (−16.9 to −6.4)	−14.9 (−20.1 to −9.8)	3.3 (−4.1 to 10.6)	.38
Month 12	−11.3 (−16.8 to −5.7)	−13.5 (−18.7 to −8.3)	2.2 (−5.4 to 9.9)	.56

Abbreviations: DCR, daily calorie restriction; HDL-C, high-density lipoprotein cholesterol; HOMA-IR, homeostasis model assessment of insulin resistance (calculated as insulin × glucose/405, where the unit of measure for insulin is in microinternational units per milliliter and the unit of measure for glucose is milligrams per deciliter); LDL-C, low-density lipoprotein cholesterol; TRE, time-restricted eating.

SI conversion factors: To convert alanine aminotransferase to microkatals per liter, multiply by 0.0167; aspartate aminotransferase to microkatals per liter, multiply by 0.0167; γ-glutamyltransferase to microkatals per liter, multiply by 0.0167; glucose to millimoles per liter, multiply by 0.0555; cholesterol (HDL-C, LDL-C, and total) to millimoles per liter, multiply by 0.0259; hemoglobin A_1c_ to proportion of total hemoglobin, multiply by 0.01; and triglycerides to millimoles per liter, multiply by 0.0113.

^a^ For the between-group difference at month 6 and at month 12 from linear mixed models that included diet, time, and diet × time interaction terms.

### Adverse Events

No deaths or serious adverse events occurred throughout the study. Occurrence of mild adverse events, including appetite change, discomfort in the stomach, constipation, dyspepsia, hunger, decreased appetite, dizziness, and fatigue, were not significantly different in the 2 groups (eTable 3 in Supplement 2).

## Discussion

This randomized clinical trial contributes novel findings on the effects of TRE vs DCR on NAFLD. First, this study indicated that the 8-hour TRE diet (eating period from 8:00 am to 4:00 pm) was no more effective in reducing the IHTG content and in achieving resolution of NAFLD among patients with NAFLD than DCR (habitual meal timing) with the same caloric intake restriction. Second, TRE and DCR diets produced comparable effects in reducing body weight, waist circumference, body fat, and visceral fat. Furthermore, both diets were equally effective in reducing blood pressure, plasma glucose level, HOMA-IR, liver enzyme levels, and lipid levels during 12 months. Third, caloric intake restriction seems to explain most of the beneficial effects of the TRE regimen.

Time-restricted eating has been promoted as a potential alternative weight loss strategy to DCR.^13,36^ However, the benefits of the TRE regimen on NAFLD are still untested or undertested in humans. Time-restricted eating regimens have either imposed a shortening window of eating while maintaining participants’ usual caloric intake^22,37^ or hypoenergetic intake.^38^ Cai et al^39^ reported that TRE with ad libitum intake did not improve liver stiffness compared with the control during a 12-week diet program among 176 patients with NAFLD. Kahleova and colleagues^23^ reported that a regimen of eating 2 meals (between 6:00 am and 4:00 pm) reduced IHTG content more than a regimen of eating 6 meals with the same caloric intake restriction in a 12-week clinical trial among 54 patients with obesity and type 2 diabetes. So far, the long-term effect of TRE on NAFLD remains uncertain.

To our knowledge, this study is the first randomized clinical trial to compare the long-term effect of TRE vs DCR on NAFLD. This trial showed that the 2 diet regimens had similar effects on reducing IHTG content and improving liver stiffness and that it was feasible for participants to adhere to their assigned calorie intake restrictions. Both diets with an energy intake of 1200 to 1800 kcal/d resulted in nearly 40% resolution of NAFLD. Furthermore, the results suggest that caloric intake restriction explained most of the beneficial effects of a TRE regimen. These findings support a strategy of TRE combined with caloric intake restriction (prescribed according to current dietary guidelines) as a viable and sustainable approach for NAFLD management.

Several small clinical trials assessed the effects of short-term TRE on weight and waist circumference in obese populations and reported inconsistent findings.^19,21,38,39,40,41^ Lowe and colleagues^41^ reported that short-term TRE had no favorable benefits on reducing body weight and waist circumference reduction among 116 adults with obesity. In contrast, Cai et al^39^ found that 12-week TRE significantly reduced body weight in 97 patients with NAFLD compared with the controls. Evidence suggests that the effect of TRE with ad libitum intake on weight loss appeared to be likely associated with a decrease in energy intake.^19,42,43^ Nevertheless, small clinical trials reported that the TRE regimens with isoenergetic intake improved body weight in healthy adults and select metabolic parameters in men with prediabetes.^22,37,40^ By contrast, another study reported no differences in body weight and waist circumference during a 12-month TRE diet program with caloric intake restriction in 58 low-income women with obesity compared with the controls.^40^ Our data indicate that both diet regimens equally reduced body weight and waist circumference and were feasible for participants to adhere to their assigned intervention in terms of energy intake restriction. However, there were no substantial differences in weight and waist circumference between TRE and DCR during the 12-month intervention. Our study suggests that long-term TRE and DCR might be equally effective and could be recommended for weight loss in individuals with obesity.

In this trial, TRE and DCR significantly reduced body fat and visceral fat with no significant between-group differences. Several small, short-term studies reported that the TRE regimen significantly reduced body fat mass.^19,40,44,45^ In contrast, de Oliveira Maranhão Pureza and colleagues^40^ compared the effect of a 12-month TRE program vs hypoenergetic diet and reported no differences in body fat in 58 women with obesity. A meta-analysis of clinical trials also suggested that TRE seems to have no favorable effect on body fat reduction compared with the controls.^36^ Our study suggests that TRE is no more effective than DCR in body fat and visceral fat reduction among individuals with obesity.

In addition, our study indicated that there were no significant differences between TRE and DCR on cardiovascular risk factors, including blood pressure, fasting glucose levels, and lipid levels. Other studies found that short-term TRE improved glycemic control, insulin sensitivity, and blood pressure in individuals with prediabetes or adults with obesity.^19,22^ By contrast, Haganes et al^45^ reported no statistically significant effect of TRE on glycemic control in women with obesity. However, these trials did not compare the effects of TRE vs DCR on metabolic risk factors in individuals with obesity. Our data showed that TRE was more effective for improving insulin sensitivity than DCR.

### Limitations

This study has limitations. First, the primary outcome was the IHTG content instead of biopsy-proven steatosis or fibrosis. However, the IHTG content measured by magnetic resonance imaging and liver stiffness measured by transient elastography are highly correlated with the histologic features of steatosis and fibrosis.^46,47^ Furthermore, physical activity was not controlled in this study because we aimed to examine isolated effects of diet intake on NAFLD. However, physical activity was assessed using the International Physical Activity Questionnaire.

## Conclusions

In this randomized clinical trial of adults with obesity and NAFLD, a TRE regimen did not achieve additional benefits for reducing IHTG content, weight, body fat, and metabolic risk factors compared with DCR, whereas TRE might be more effective in improving insulin sensitivity than DCR. In addition, both diets produced a comparable effect on liver stiffness and resolution of NAFLD. These data support the importance of caloric intake restriction when adhering to a regimen of TRE for the management of NAFLD.

## Notes

Supplement 1.
Trial Protocol and Statistical Analysis Plan

Supplement 2. **eTable 1.** Diet Adherence and Physical Activity During Intervention **eTable 2.** Sensitivity Analyses of the Main Outcomes **eTable 3.** Adverse Events During Intervention **eFigure 1.** Effect of Diets on the Intrahepatic Triglyceride (IHTG) Content by Subgroups of Adherence **eFigure 2.** Effect of Time-Restricted Eating Versus Daily Calorie Restriction on the Weight and Waist Circumference

Supplement 3.
Data Sharing Statement
