# Supplementary material for: Effects of Time-Restricted Eating on Nonalcoholic Fatty Liver Disease: The TREATY-FLD Randomized Clinical Trial
Source: JAMA Netw Open. 2023 Mar 17;6(3):e233513. doi: 10.1001/jamanetworkopen.2023.3513 (PMC10024204; doi:10.1001/jamanetworkopen.2023.3513)
Supplement: Supplement 1. — Trial Protocol and Statistical Analysis Plan [file jamanetwopen-e233513-s001.pdf]

This supplement contains the following items:

1. **Original protocol (Page 2-14), final protocol (Page 15-28), summary of changes (Page 29)**
2. **Statistical analysis plan (the only version) (Page 30-31)**

1. Original protocol, final protocol, summary of changes

1.1 Original protocol

Effects of Time-Restricted Eating on Nonalcoholic Fatty Liver Disease

(TREATY-FLD Study)

Study Protocol

Nanfang Hospital of Southern Medical University, Guangzhou, China

ABBREVIATIONS

AE: Adverse events
ALT: Alanine transaminase
AST: Aspartate aminotransferase
BMI: Body mass index
CER: Continuous energy restriction
CT: Computer tomography
DEXA: Dual energy X-ray assessment
DCR: Daily calorie restriction
HbA1c: glycated hemoglobin
HDL-c: High-density lipoprotein cholesterol
IF: Intermittent Fasting
IHTG: Intrahepatic triglyceride content
LDL-c: Low-density lipoprotein cholesterol
MRI: Magnetic resonance imaging
NAFLD: Nonalcoholic fatty liver disease
OGTT: oral glucose tolerance test
PWV: Pulse wave velocity
TRE: Time-restricted Eating

1. Background

Nonalcoholic fatty liver disease (NAFLD) has become one of the most challenging public health problems. The prevalence of NAFLD is high and increasing with the improvement of living standard and changes in lifestyle in recent years. It affects 10 - 45% of the general population and 60 - 70% of obese adults in western countries (*1, 2*). NAFLD represents excessive accumulation of triglycerides (TG) the liver. Due to synthesis of TG in hepatocytes, excessive TG are stored in the liver and lead to hepatic steatosis. It can progress to steatohepatitis, cirrhosis or even liver cancer (*3-5*). Furthermore, NAFLD is considered a risk factor for type 2 diabetes and cardiovascular disease (*6*).

Diet, sedentary lifestyle, and genetic predisposition have been associated with increased risk of NAFLD (*6*). With the progressive global epidemic of obesity, it is expected with increased risk of NAFLD will rapidly increase. Previous studies indicated that obesity was a major risk for NAFLD, and the prevalence of NAFLD among subjects with BMI > 30 kg/m^2 is four times higher than that among normal-weight subjects (*7*). Obesity is closely related to insulin resistance. Furthermore, it is associated with hyperinsulinemia and high inflammatory cytokine levels, which can promote the development of hepatocyte steatosis (*8, 9*). On the other hand, NAFLD is considered to be a manifestation of metabolic syndrome, and is an independent risk factor for diabetes and cardiovascular disease (*6, 10-12*). Therefore, seeking an optimal treatment for NAFLD and related metabolic disorders has become a hot topic for researchers.

Lifestyle interventions play a role in improving NAFLD by reducing weight and insulin resistance (*13*). Currently the most common form of dietary regimens is continuous energy restriction (CER), a daily caloric restriction (DCR) by at least 30% or by approximately 750-1,000 kcal/day, which has been proven to be effective in reducing hepatic fat accumulation and serum liver enzyme levels when achieving a 5% weight loss among patients with NAFLD (*14*). However, it is challenging to maintain the weight with the decline of compliance to a calorie restricted diet and persistence of hormonal adaptation is associated with slow weight regain over the long term (*15-18*) .

Time-restricted eating (TRE) is a specific intermittent fasting (IF) regimen involving confining the eating window from 4 to12 hours within a 24-hour cycle (*19, 20*). Prior studies reported that TRE can result in a reduction intrahepatic fat content. Hodge et al conducted a 12-week pilot study in 32 NAFLD patients and found that withholding caloric intake for 16 hours (8:00 PM to 12:00 PM the following day) significantly improved liver stiffness and hepatic steatosis measured by transient elastography compared to standard care (*21*).A prior small clinical trial reported that the regimen of eating two meals (eating periods from 6:00 AM to 4:00 PM) reduced intrahepatic lipids measured by proton magnetic resonance spectroscopy compared with the control (eating six smaller meals) among 54 patients with type 2 diabetes during 12 weeks intervention (*22*). In addition, TRE was a feasibility strategy and accessibly to enhance adherence in previous studies (*23-26*). However, previous studies will be small and short-term, the efficacy of TRE on NAFLD remains uncertain. Furthermore, these studies did not compare the effects of TRE versus DCR on intrahepatic lipids in patients with NAFLD.

2. Specific Aims

We propose to conduct a randomized controlled trial to compare the effects of TRE versus DCR on the intrahepatic triglyceride (IHTG) content and metabolic risk factors among obese individuals with NAFLD. We hypothesize that TRE would be more effective than DCR in improving NAFLD and metabolic risk factors.

3. Research Design and Methods

3.1 Overall designs

This is a randomized, group-paralleled, observer-masked clinical trial. After screen, all eligible subjects will be randomly assigned to the TRE group or DCR group with an allocation ratio of 1:1 (Figure 1). Participants assigned to the TRE group will be instructed to eat only from 8:00 AM to 4:00 PM and only noncaloric beverages will be permitted outside of the eating window over 6 months. Participants in the DCR group will be instructed to follow a calorie restriction diet without restricting eating period for 6 months.

Figure 1. The procedure for the trial

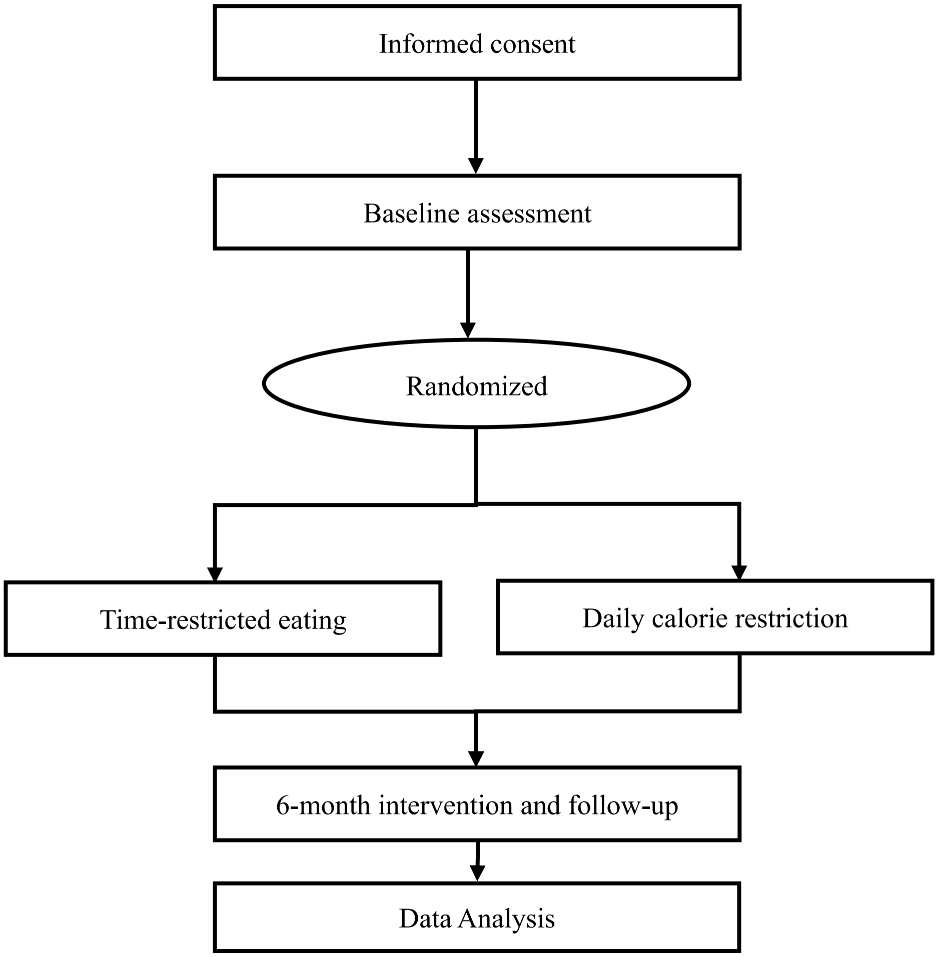

3.2 Statistical power and sample size

The study will enroll 86 participants, who will be randomly allocated to TRE or DCR group with an allocation ratio of 1:1. The sample size calculation is based on the primary outcome(*22, 27*), change of IHTG content at 6-month and 12-month assessment. We anticipate 90% statistical power to detect a significant difference of 0.8% in IHTG content (SD, 1.0%) between the TRE group and the DCR group at a significance level of 0.05 using a 2-tailed test. Thus, 34 subjects in each group are required. Assuming a dropout rate of 20%, we will recruit 43 subjects to each group in the proposed clinical trial.

4. Inclusion and Exclusion criteria

4.1 Inclusion criteria

- Men or women aged ≥ 18 years;
- Subjects with NAFLD determined by MRI (intrahepatic triglyceride content ≥ 5%);
- Body mass index (BMI) of 28.0 to 45.0 kg/m2

4.2 Exclusion criteria:

- History of alcoholic liver disease, chronic viral hepatitis, drug-induced hepatitis, autoimmune hepatitis, cirrhosis, and liver cancer;
- History of HIV, or active pulmonary tuberculosis;
- Diagnosis of type 1 and type 2 diabetes;
- History of malignant tumors;
- Serious liver dysfunction or chronic kidney disease (AST or ALT > 3 times the upper limit of normal, or eGFR < 30 ml/min/1.73 m2);
- Significant alcohol consumption in the past six months (Consumed more than 20 g/day for women or 30 g/day for men);
- History of serious cardiovascular or cerebrovascular disease (angina, myocardial infarction or stroke) in the past 6 months;
- History of severe gastrointestinal diseases or gastrointestinal surgery in the past 12 months;
- History of Cushing's syndrome, hypothyroidism, acromegaly, hypothalamic obesity;
- Being a smoker or having been a smoker in the 3 months prior to their screening visit;
- Taking medications affecting weight or energy intake/energy expenditure in the last 6 months, including weight loss medications, antipsychotic drugs or other medications as determined by the study physician;
- Currently participating in weight loss programs or weight change in the past 3 months (> 5% current body weight);
- Women who are pregnant or plan to become pregnant;
- Patients who cannot be followed for 24 months (due to a health situation or migration);
- Patients who are unwilling or unable to give informed consent.

5. Randomization and masking

Randomization will be conducted by the Nanfang hospital of Southern Medical University in Guangzhou, China. The randomization scheme is generated with SAS PROC PLAN (SAS Institution Inc). Although this is an open-label trial, the research assistants who collect study outcome data will be masked to participants' intervention assignment.

6. Intervention

This trial aims to compare the effect of TRF versus CER on the improvement of NAFLD and metabolic risk factors. The duration of the intervention is 6 months.

6.1 Diet protocols

All participants will be instructed to follow a diet of 1500-1800kcal/d for men and 1200-1500kcal/d for women (40-55% of energy as carbohydrate, 15-20% as protein, 20-30% as fat) (*28*). Participants assigned to the TRE group will be instructed to eat from 8:00 AM to 4:00 PM and only noncaloric beverages is permitted outside of the eating window. Participants in the DCR group will be instructed to follow a calorie restriction diet without restricting eating period. All participants will be provided with one protein shake (Nutriease, Zhejiang Nutriease Co. Ltd., China) (Table 1) per day and receive dietary counseling for the duration of the study. All participants will receive written dietary information booklets with portion advice and sample menus and have similar dietary energy restrictions in accordance with Dietary Guidelines for macronutrient intake (*28, 29*).

Table 1. Nutrition Information of protein shake

**Items**	**per serving 28g**	**NRV%**
Energy	451 kJ	5%
Protein	14.4 g	24%
Fat	1.9 g	3%
Trans fat	0 g	

Carbohydrate	7.2 g	2%
Dietary Fiber	1.6 g	6%
Sodium	203 mg	10%
Vitamin A	224 μg RE	28%
Vitamin D	0.3 μg	6%
Vitamin E	3.36 mg α-TE	24%
Vitamin B1	0.39 mg	28%
Vitamin B2	0.39 mg	28%
Vitamin B6	0.39 mg	28%
Vitamin B12	0.70 μg	29%
Vitamin C	39.2 mg	39%
Nicotinic acid	3.92 mg	28%
Folic acid	112 μg DFE	28%
Pantothenic acid	1.26 mg	25%
Magnesium	36 mg	12%
Calcium	84 mg	11%
Iron	3.4 mg	23%
Zinc	2.80 mg	19%

6.2 Intervention monitoring

Participants will be encouraged to weigh foods to ensure accuracy of intake. All participants will be required to write their dietary log and record daily food picture and mealtime on a custom mobile study application (App), which will be reviewed daily by study nutritionist. Caloric intake assessment will be conducted by the nutritionist based on each participant's log and daily food photos based on the nutrient content listed in the China Food Composition Table (*30*). Intervention participants will receive follow-up telephone calls or App message twice per week, and meet with the dietician or nutritionist individually every 2 weeks to assess their adherence to the program and provide suggestions for improvements and personalized energy targets (Table 2).

Table 2. Frequency of Intervention monitoring

	**Months 1 - 3**	**Months 4 - 6**
24-hour dietary log	Everyday	Everyday
Telephone or app message delivered guidance	Twice per week	Twice per week
Face to face counselling	Twice to three times per month	Twice per month

6.3 Support and education

All participants will be instructed to attend health education sessions monthly. Education sessions content will include weight measurement, dietary recall, dietary instruction (e.g., recipes, sample menus, food list, knowledge about macronutrients counting, nutrition labels reading), behavioral strategies, general health knowledge of NAFLD and metabolic disease (Table 3). All participants will be instructed not to change their physical activity habits throughout the trial.

Table 3. Topics of health education sessions

	Topics
Behavioral strategies	Introduction of exercise knowledge, guidance of exercise methods, improvement of sleep, etc.
Dietary instruction	Portions of food, knowledge about macronutrients counting, nutrition labels reading, etc.
General health knowledge	Causes and harms of NAFLD, etc.

7. Study outcomes

7.1 Primary outcome

Changes in the IHTG content from baseline at 6-month assessment.

7.2 Secondary outcomes

1) Change in liver stiffness assessed by transient elastography (Fibroscan)
2) Change in body weight, waist circumference, and body fat composition
3) Change in cardiovascular risk factors (Blood pressure, lipids, glucose)
4) Change in insulin resistance and β cell function
5) Change in liver enzymes
6) Change in pulse wave velocity (PWV)
7) Change in Depression, quality of sleep, quality of life

8. Participant Termination Criteria

1） Occurrence of serious security issue; serious, unexpected adverse events that are directly related to the intervention.
2） Pregnancy;
3） Subjects ask to withdraw the informed consent form;
4） Unwillingness to compliant to the intervention or other unexpected reasons.

9. Data collection

9.1 Questionnaire

Medical history, personal information (age, gender, education, household income, etc.), lifestyle risk factors (cigarette smoking, alcohol drinking), physical activity, three 24-hour dietary recall, sleep quality, and quality of life.

9.2 Anthropometric measurements:

Height, weight, waist circumference, blood pressure, heart rate.

9.3 Biochemical measurements:

Overnight blood samples and spot urine will be collected to measure metabolic risk factors (fasting plasma glucose, insulin, glycosylated hemoglobin (HbA1c), serum triglycerides, total cholesterol, LDL- and HDL-cholesterol, liver enzymes, serum creatinine and urine acid. Oral glucose tolerance test (OGTT) measurement of blood glucose and serum insulin (0’, 30’,120’ min).

9.4 Transient elastography:

Transient elastography (FibroScan 502 Touch, Echosens) will be used to measure liver stiffness.

9.5 Body composition:

Whole body compositions will be quantified using a whole-body dual x-ray system (Lunar iDXA, GE Healthcare).

9.6 Body fat rate:

The bioelectrical impedance method will be used to measure body fat rate using the human body fat analyzer (V.Body HBF-371, Omron).

9.7 Visceral and subcutaneous fat area

Visceral and subcutaneous fat area will be measured using abdominal CT (Revolution, GE Healthcare) at the level of the lumbar vertebra(*31*).

9.8 The IHTG content:

The IHTG content will be measured using magnetic resonance imaging (Ingenia 3.0T mDIXON Quant, Philips Healthcare). Regions of interest (ROIs) will be selected in in the left liver lobe, the middle of the right liver lobe, and the posterior portion of the right liver lobe in the transverse sections through the right hepatic portal vein and below the second hepatic portal vein(*32-34*). All the selected ROIs should include hepatic parenchyma only, and exclude any biliary, vascular, or extrahepatic structures. The IHTG content is calculated as the mean of values from five ROIs.

10. Follow-up visits

The time points for baseline and outcome measurements are shown in Table 4.

Table 4. Follow-up Plan

	**M0**	**M1**	**M2**	**M3**	**M4**	**M5**	**M6**
**Informed consent**	X						
**Randomization**	X						
**Demographic information**	X						
**Medical history and medications**	X			X			X
**Lifestyle questionnaire**	X			X			X
**24-hour dietary recalls *3 days**	X						X
**Physical and biochemical examinations**							
Weight, waist circumference	X	X	X	X	X	X	X
Blood pressure, heart rate	X	X	X	X	X	X	X
Blood glucose, HbA1c	X			X			X
Lipids	X			X			X
OGTT + insulin (0', 30',120' min)	X						X
Liver enzymes,<br>Urine acid and creatinine	X			X			X
**Examinations**							
Liver MRI	X						X
Transient elastography	X						X
Abdominal CT	X						X
DEXA	X						X
PWV	X						X
Body fat rate (BI)	X	X	X	X	X	X	X
**Blood, urine and feces sample**	X						X
**Adverse event**	X	X	X	X	X	X	X
**Protein Shake**		X	X	X	X	X	X
**Diet counseling and health education**		X	X	X	X	X	X

11. Safety and adverse events

11.1 Definition of adverse events

Adverse events (AE) are defined as the occurrence of new diagnosis or deterioration of diseases during the treatment. The causal

relationship to the intervention will be further evaluated.

11.2 Definition of serious adverse events

Severe adverse events (SAE) are defined as the occurrence of any acute life-threatening event, a hospitalization for any cause other than routine delivery, prolonged or permanent disability.

11.3 Surveillance and recording

Protection of participants from risks related to the intervention is of paramount concern to the physicians. All participants will be evaluated for their medical history before enrollment. Contact information of the physicians are available for all the participants.

All participants should be contacted regularly to evaluate the occurrence, severity, and duration of physical discomfort, including dizziness, fatigue, abdominal pain, headache, appetite change, constipation, or other adverse events during intervention. Evidence of the occurrence of adverse events should be based on participants' self-report rather than suggestive questioning. Any medical adverse events will be recorded and the nature, severity, and cause-effect relationship will be evaluated by physicians and recorded in a timely manner on case report forms.

11.4 Assessment of severity

The severity of adverse events is assessed as follows:

Mild: usually temporary, and not affecting daily activities;

Moderate: causing discomfort and affecting normal activities, thought tolerable and not requiring participants to take any medication.

Severe: disrupting daily activities and intolerable, requiring participants to take medication immediately.

With regard to the causal-effect relationship, medical adverse events are divided into five categories: definite, probable, possible, unlikely, and unrelated. Only "definite" and "probable" are counted as adverse events.

11.5 Treatment

Any adverse events, appropriate care or medical treatment (if necessary) should be immediately provided to keep the safety of all participants. In addition, all adverse events and serious adverse events and treatment should be recorded and reported to the Principal Investigator and the Committee within 24 hours and complete the case report form.

12. Data management

12.1. Case report form

All case report forms for each participant should be filled out by study staff in a timely manner. The case report form should be double-checked for potential errors or missing data prior to patients leaving the clinic. All data, including screening assessments, questionnaires, physical examinations, and laboratory examinations, will be filed in the participant's chart. Original documents, participants' charts, and CRF forms will be stored in the study office.

12.2. Data entry

All data will be double-entered by researcher staff. Two sets of databases will be generated and tested for consistency using the SAS program. Whenever inconsistencies are found, the data will be corrected by re-examination of the original case report forms or laboratory reports.

12.3 Data reports

Several standardized reports will be generated as follows: 1) participant recruitment and follow-up; 2) demographics; 3) data quality and monitoring; 4) adverse events. These reports will be used for study management. These reports will be blinded to research personnel who collect study outcomes.

13. Data analysis methods

Data will be analyzed according to participants' randomization assignments, regardless of their subsequent status (intention-to-

treat). PROC MIXED of SAS statistical software, version 9.4 (SAS Institute Inc), will be used to obtain point estimates and SEs of the treatment effects and to test for differences between treatments. Group differences in the study outcomes will be evaluated using the general linear model for continuous variables and the chi-squared test for categorical variables. A mixed-effects model will be used to assess the effects of diet programs on the change in IHTG content and main outcomes and an autoregressive correlation matrix will be used to correct within-participant correlation for repeated measurements. In this model, participants will be assumed to be random effects, and intervention group, time, and their 2-factor interactions will be assumed to be estimable fixed effects. Multiple imputations for missing data in the multivariable analyses will be conducted using the Markov chain Monte Carlo method. Data will be presented as least-squares means with 95% confidence intervals (CIs). $P < 0.05$ will be considered statistically significant.

14. Training for study staff

All study staff (physicians and nurses) will be trained and certified before the study is initiated. The training program will include instruction on questionnaire administration and anthropometrics (height, weight, waist circumference, and hip girth) and blood pressure measurement. A standard questionnaire will be used, which includes personal information (name, sex, age, place of birth, marital status, occupation, education, household income, etc.), medical history, medication use, family history, lifestyle factors (smoking and drinking), physical activity, dietary habits, and menstrual and reproductive history. Anthropometric measurements and blood pressure will be obtained using a standard protocol. The data, including that for exercise intensity, duration, heart rate, and anthropometric measurements, will be collected and recorded in CRFs by nurses every week. All technologists are experienced and masked to participant randomization.

15.Quality Control

China's Standard for Quality Management of Clinical Trials (GCP) and other relevant laws and regulations on clinical trials will be complied with. The standard quality control process will be carried out at each step of the study, including repeated laboratory measurements in 5-10% of random blind samples, and all data will be entered independently.

Establish a research procedure manual (MOP): we will develop a standardized process of data collection, which will include the recruitment of subjects, instructions for the use of tables, intervention programs, index measurement, specimen collection and management, etc., to guide all tabular information entry and diagnosis and treatment processes, and other aspects of the study.

Training: all researchers will receive relevant training before the start of the study, including standardized processes, recruitment processes, follow-up, intervention programs, measurement procedures, and the use of MOP. At the same time, regular retraining sessions will be conducted to ensure the quality of research.

Quality monitoring and reporting: quality control personnel will regularly review the QC data, including the timeliness of the completion of the visit, data collection, program implementation and data quality.

16. Organization and Communication

A steering committee (Steering committee, SC), chaired by Drs Huijie Zhang and Yikai Xu, Nanfang Hospital, Southern Medical University, will be responsible for the study. The research team consists of researchers and health management team members from the Norte Health Management Center. the steering committee (SC) is responsible for organizing and supervising research implementation, approving the research program, including modification of the research program, monitoring the recruitment and withdrawal process of subjects, implementation of intervention programs, recruitment and intervention of subjects, data collection and quality control, and responding to questions raised by the ethics body review committee (Institutional review board, IRB), etc. The research center will be involved in the recruitment, intervention, data collection and quality control of the subjects. the research center will do randomization and data analysis, and help prepare to report research results and papers for publication. In order to facilitate communication between investigators and researchers, regular working meetings will be held. SC will discuss relevant scientific and management issues in the study with three live meetings a year and monthly conference calls. Among them, important information, such as recruitment progress,

data completion and quality, and compliance with intervention programs, will be sent monthly by e-mail to committee members and key staff.

17. Ethics Approval

This clinical trial must comply with the Helsinki Declaration (1996 edition) and the relevant regulations of Chinese clinical trials. Before the trial begins, the hospital ethics committee of the research unit shall review the trial plan and issue the approval document before the implementation of the trial plan.

For all research program amendments (excluding administrative amendments and amendments that have no impact on the implementation of subjects, data or trials), the amendment and the applicable informed consent must be submitted to the Ethics Committee for approval immediately prior to the implementation of these changes. The researcher is responsible for ensuring that the conditions approved by the study are met and that the trial correction program or serious adverse events are reported to the ethics committee or the corresponding organization as required by the ethics committee.

All subjects will be given the opportunity to read the entire informed consent document and, by the researcher or authorized researcher, to provide the subjects, in writing, with a complete and comprehensive presentation of the background, purpose, research methods, research process, test items to be involved, individual rights and obligations, possible risks, and possible benefits of the research process. The subjects had enough time to ask questions, and the researchers needed to provide truthful and accurate answers in plain language. The subjects had sufficient time to discuss with other family members whether to participate in the study. The subjects should know that they can withdraw from the study at any time without any reason; the subjects agree to collect and apply the data related to the study and are willing to cooperate with the follow-up. When they exit, the subjects are asked whether they agree to apply the collected data. Participants will be required to sign informed consent before they can be selected for clinical trials. Informed consent shall be kept for reference as one of the original materials for clinical trials.

18. Proposed study timeline

The clinical study is expected to take about 3 years after registration in the clinicaltrials.gov (Identifier: NCT03786523), the trial is expected to take about 3 years to carry out this clinical study, in which MOP development and personnel training and subject recruitment time is 1 year. Intervention and data collection cycle is 6 months, data entry and analysis and paper writing lasted about 0.5 years.

References

1. J. D. Browning *et al.*, Prevalence of hepatic steatosis in an urban population in the United States: impact of ethnicity. *Hepatology* **40**, 1387-1395 (2004).
2. C. D. Williams *et al.*, Prevalence of nonalcoholic fatty liver disease and nonalcoholic steatohepatitis among a largely middle-aged population utilizing ultrasound and liver biopsy: a prospective study. *Gastroenterology* **140**, 124-131 (2011).
3. C. Garcia-Monzon *et al.*, Characterization of pathogenic and prognostic factors of nonalcoholic steatohepatitis associated with obesity. *Journal of hepatology* **33**, 716-724 (2000).
4. P. Gupte *et al.*, Non-alcoholic steatohepatitis in type 2 diabetes mellitus. *Journal of gastroenterology and hepatology* **19**, 854-858 (2004).
5. G. Musso, R. Gambino, M. Cassader, G. Pagano, Meta-analysis: natural history of non-alcoholic fatty liver disease (NAFLD) and diagnostic accuracy of non-invasive tests for liver disease severity. *Annals of medicine* **43**, 617-649 (2011).
6. G. Targher, C. P. Day, E. Bonora, Risk of cardiovascular disease in patients with nonalcoholic fatty liver disease. *The New England journal of medicine* **363**, 1341-1350 (2010).
7. P. Haentjens *et al.*, Identifying non-alcoholic fatty liver disease among asymptomatic overweight and obese individuals by clinical and biochemical characteristics. *Acta clinica Belgica* **64**, 483-493 (2009).
8. J. C. Cohen, J. D. Horton, H. H. Hobbs, Human fatty liver disease: old questions and new insights. *Science* **332**, 1519-1523 (2011).
9. A. M. Cali *et al.*, Intrahepatic fat accumulation and alterations in lipoprotein composition in obese adolescents: a perfect proatherogenic state. *Diabetes care* **30**, 3093-3098 (2007).
10. L. Shen, Treatment implication from the potential association between nonalcoholic fatty liver disease and cardiovascular disease. *Hepatology* **54**, 377-378 (2011).
11. J. B. Schwimmer, P. E. Pardee, J. E. Lavine, A. K. Blumkin, S. Cook, Cardiovascular risk factors and the metabolic syndrome in pediatric nonalcoholic fatty liver disease. *Circulation* **118**, 277-283 (2008).
12. M. Hamaguchi *et al.*, The metabolic syndrome as a predictor of nonalcoholic fatty liver disease. *Annals of internal medicine* **143**, 722-728 (2005).
13. M. Romero-Gomez, S. Zelber-Sagi, M. Trenell, Treatment of NAFLD with diet, physical activity and exercise. *Journal of hepatology* **67**, 829-846 (2017).
14. N. Chalasani *et al.*, The diagnosis and management of nonalcoholic fatty liver disease: Practice guidance from the American Association for the Study of Liver Diseases. *Hepatology* **67**, 328-357 (2018).
15. P. Sumithran *et al.*, Long-term persistence of hormonal adaptations to weight loss. *The New England journal of medicine* **365**, 1597-1604 (2011).
16. G. Diabetes Prevention Program Research *et al.*, 10-year follow-up of diabetes incidence and weight loss in the Diabetes Prevention Program Outcomes Study. *Lancet* **374**, 1677-1686 (2009).
17. A. R. G. Look, Eight-year weight losses with an intensive lifestyle intervention: the look AHEAD study. *Obesity* **22**, 5-13 (2014).
18. K. R. Middleton, S. D. Anton, M. G. Perri, Long-Term Adherence to Health Behavior Change. *American journal of lifestyle medicine* **7**, 395-404 (2013).
19. R. V. Seimon *et al.*, Do intermittent diets provide physiological benefits over continuous diets for weight loss? A systematic review of clinical trials. *Molecular and cellular endocrinology* **418 Pt 2**, 153-172 (2015).
20. M. P. Mattson, V. D. Longo, M. Harvie, Impact of intermittent fasting on health and disease processes. *Ageing Res Rev* **39**, 46-58 (2017).
21. A. Hodge *et al.*, Non-alcoholic fatty liver disease Intermittent Fasting Time Intervention (NIFTI): Fasting without calorie

restriction improves hepatic transient elastography, visceral adiposity and insulin resistance compared to standard care. *Hepatology* **60**, 632a-632a (2014).

22. H. Kahleova *et al.*, Eating two larger meals a day (breakfast and lunch) is more effective than six smaller meals in a reduced-energy regimen for patients with type 2 diabetes: a randomised crossover study. *Diabetologia* **57**, 1552-1560 (2014).
23. E. F. Sutton *et al.*, Early Time-Restricted Feeding Improves Insulin Sensitivity, Blood Pressure, and Oxidative Stress Even without Weight Loss in Men with Prediabetes. *Cell metabolism* **27**, 1212-1221 e1213 (2018).
24. S. Gill, S. Panda, A Smartphone App Reveals Erratic Diurnal Eating Patterns in Humans that Can Be Modulated for Health Benefits. *Cell metabolism* **22**, 789-798 (2015).
25. G. M. Tinsley *et al.*, Time-restricted feeding in young men performing resistance training: A randomized controlled trial. *European journal of sport science* **17**, 200-207 (2017).
26. J. D. LeCheminant, E. Christenson, B. W. Bailey, L. A. Tucker, Restricting night-time eating reduces daily energy intake in healthy young men: a short-term cross-over study. *The British journal of nutrition* **110**, 2108-2113 (2013).
27. H. Kahleova *et al.*, Erratum to: Eating two larger meals a day (breakfast and lunch) is more effective than six smaller meals in a reduced-energy regimen for patients with type 2 diabetes: a randomised crossover study. *Diabetologia* **58**, 205 (2015).
28. R. H. Eckel *et al.*, 2013 AHA/ACC guideline on lifestyle management to reduce cardiovascular risk: a report of the American College of Cardiology/American Heart Association Task Force on Practice Guidelines. *J Am Coll Cardiol* **63**, 2960-2984 (2014).
29. C. N. Society, *Dietary Guidelines for Chinese Residents*. (People's Medical Publishing House, Beijing, China, ed. 1, 2016).
30. Y. Yang, *China Food Composition Tables Standard Edition*. (Beijing University Medical Press, Beijing, China, ed. 6, 2018).
31. Y. Tong, J. K. Udupa, D. A. Torigian, Optimization of abdominal fat quantification on CT imaging through use of standardized anatomic space: a novel approach. *Medical physics* **41**, 063501 (2014).
32. S. D. Serai, J. R. Dillman, A. T. Trout, Proton Density Fat Fraction Measurements at 1.5- and 3-T Hepatic MR Imaging: Same-Day Agreement among Readers and across Two Imager Manufacturers. *Radiology* **284**, 244-254 (2017).
33. Y. Zhang *et al.*, Comparison of CT and magnetic resonance mDIXON-Quant sequence in the diagnosis of mild hepatic steatosis. *The British journal of radiology* **91**, 20170587 (2018).
34. G. M. Kukuk *et al.*, Comparison between modified Dixon MRI techniques, MR spectroscopic relaxometry, and different histologic quantification methods in the assessment of hepatic steatosis. *European radiology* **25**, 2869-2879 (2015).

1.2 Final Protocol

Effects of Time-Restricted Eating on Nonalcoholic Fatty Liver Disease

(TREATY-FLD Study)

Study Protocol

Nanfang Hospital of Southern Medical University, Guangzhou, China

ABBREVIATIONS

AE: Adverse events
ALT: Alanine transaminase
AST: Aspartate aminotransferase
BMI: Body mass index
CER: Continuous energy restriction
CT: Computer tomography
DEXA: Dual energy X-ray assessment
DCR: Daily calorie restriction
HbA1c: glycated hemoglobin
HDL-c: High-density lipoprotein cholesterol
IF: Intermittent Fasting
IHTG: Intrahepatic triglyceride content
LDL-c: Low-density lipoprotein cholesterol
MRI: Magnetic resonance imaging
NAFLD: Nonalcoholic fatty liver disease
OGTT: oral glucose tolerance test
PWV: Pulse wave velocity
TRE: Time-restricted Eating

1. Background

Nonalcoholic fatty liver disease (NAFLD) has become one of the most challenging public health problems. The prevalence of NAFLD is high and increasing with the improvement of living standard and changes in lifestyle in recent years. It affects 10 - 45% of the general population and 60 - 70% of obese adults in western countries (*1, 2*). NAFLD represents excessive accumulation of triglycerides (TG) the liver. Due to synthesis of TG in hepatocytes, excessive TG are stored in the liver and lead to hepatic steatosis. It can progress to steatohepatitis, cirrhosis or even liver cancer (*3-5*). Furthermore, NAFLD is considered a risk factor for type 2 diabetes and cardiovascular disease (*6*).

Diet, sedentary lifestyle, and genetic predisposition have been associated with increased risk of NAFLD (*6*). With the progressive global epidemic of obesity, it is expected with increased risk of NAFLD will rapidly increase. Previous studies indicated that obesity was a major risk for NAFLD, and the prevalence of NAFLD among subjects with BMI > 30 kg/m^2 is four times higher than that among normal-weight subjects (*7*). Obesity is closely related to insulin resistance. Furthermore, it is associated with hyperinsulinemia and high inflammatory cytokine levels, which can promote the development of hepatocyte steatosis (*8, 9*). On the other hand, NAFLD is considered to be a manifestation of metabolic syndrome, and is an independent risk factor for diabetes and cardiovascular disease (*6, 10-12*). Therefore, seeking an optimal treatment for NAFLD and related metabolic disorders has become a hot topic for researchers.

Lifestyle interventions play a role in improving NAFLD by reducing weight and insulin resistance (*13*). Currently the most common form of dietary regimens is continuous energy restriction (CER), a daily caloric restriction (DCR) by at least 30% or by approximately 750-1,000 kcal/day, which has been proven to be effective in reducing hepatic fat accumulation and serum liver enzyme levels when achieving a 5% weight loss among patients with NAFLD (*14*). However, it is challenging to maintain the weight with the decline of compliance to a calorie restricted diet and persistence of hormonal adaptation is associated with slow weight regain over the long term (*15-18*) .

Time-restricted eating (TRE) is a specific intermittent fasting (IF) regimen involving confining the eating window from 4 to12 hours within a 24-hour cycle (*19, 20*). Prior studies reported that TRE can result in a reduction intrahepatic fat content. Hodge et al conducted a 12-week pilot study in 32 NAFLD patients and found that withholding caloric intake for 16 hours (8:00 PM to 12:00 PM the following day) significantly improved liver stiffness and hepatic steatosis measured by transient elastography compared to standard care (*21*).A prior small clinical trial reported that the regimen of eating two meals (eating periods from 6:00 AM to 4:00 PM) reduced intrahepatic lipids measured by proton magnetic resonance spectroscopy compared with the control (eating six smaller meals) among 54 patients with type 2 diabetes during 12 weeks intervention (*22*). In addition, TRE was a feasibility strategy and accessibly to enhance adherence in previous studies (*23-26*). However, previous studies will be small and short-term, the efficacy of TRE on NAFLD remains uncertain. Furthermore, these studies did not compare the effects of TRE versus DCR on intrahepatic lipids in patients with NAFLD.

2. Specific Aims

We propose to conduct a randomized controlled trial to compare the effects of TRE versus DCR on the intrahepatic triglyceride (IHTG) content and metabolic risk factors among obese individuals with NAFLD. We hypothesize that TRE would be more effective than DCR in improving NAFLD and metabolic risk factors.

3. Research Design and Methods

3.1 Overall designs

This is a randomized, group-paralleled, observer-masked clinical trial. After screen, all eligible subjects will be randomly assigned to the TRE group or DCR group with an allocation ratio of 1:1 (Figure 1). The duration of intervention included the original designed 6 months and the next 6 months follow-up visits. Participants assigned to the TRE group will be instructed to eat only from 8:00 AM to 4:00 PM and only noncaloric beverages will be permitted outside of the eating window during the initial 6-month weight-loss phase and the next 6-month follow-up. Participants in the DCR group will be instructed to follow a calorie restriction diet without

restricting eating period for 12 months.

Figure 1. The procedure for the trial

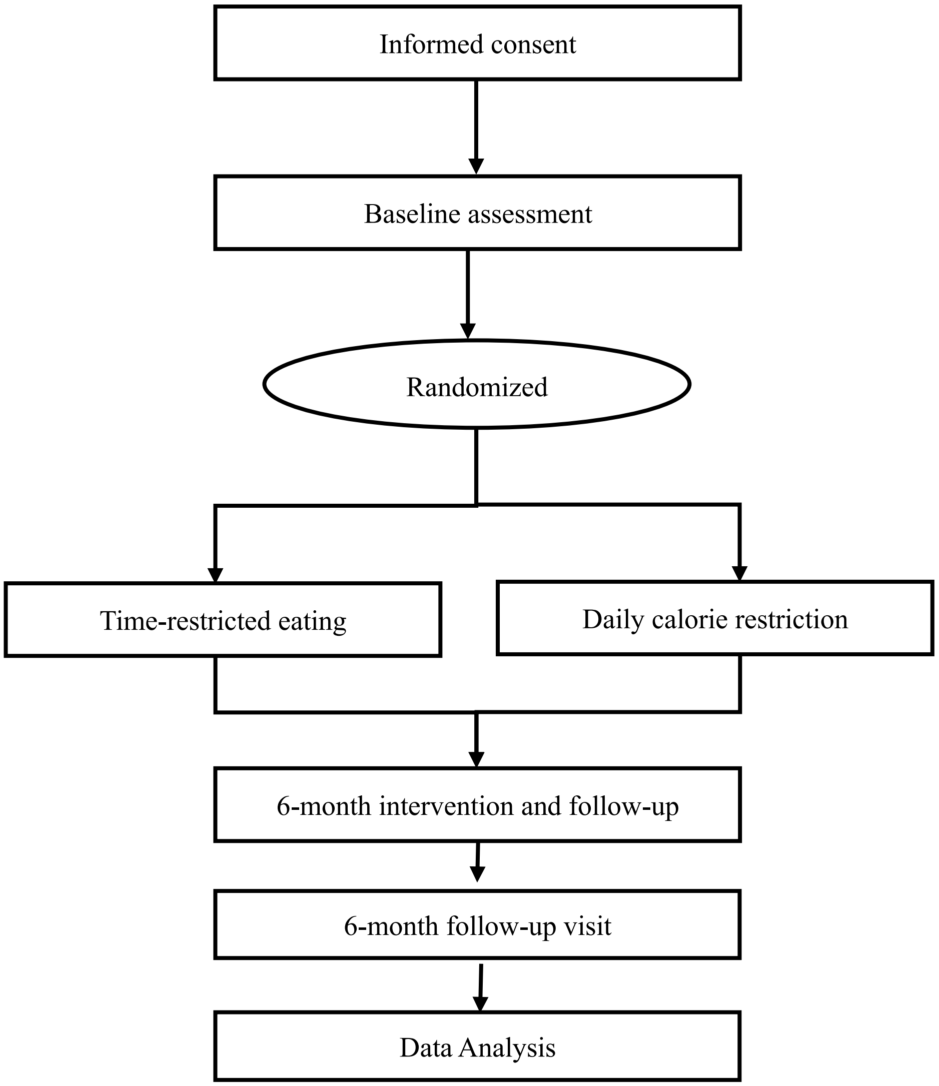

3.2 Statistical power and sample size

The study will enroll 86 participants, who will be randomly allocated to TRE or DCR group with an allocation ratio of 1:1. The sample size calculation is based on the primary outcome(*22, 27*), change of IHTG content at 6-month and 12-month assessment. We anticipate 90% statistical power to detect a significant difference of 0.8% in IHTG content (SD, 1.0%) between the TRE group and the DCR group at a significance level of 0.05 using a 2-tailed test. Thus, 34 subjects in each group are required. Assuming a dropout rate of 20%, we will recruit 43 subjects to each group in the proposed clinical trial.

4. Inclusion and Exclusion criteria

4.1 Inclusion criteria

- Men or women aged ≥ 18 years;

- Subjects with NAFLD determined by MRI (intrahepatic triglyceride content ≥ 5%);
- Body mass index (BMI) of 28.0 to 45.0 kg/m2

4.2 Exclusion criteria:

- History of alcoholic liver disease, chronic viral hepatitis, drug-induced hepatitis, autoimmune hepatitis, cirrhosis, and liver cancer;
- History of HIV, or active pulmonary tuberculosis;
- Diagnosis of type 1 and type 2 diabetes;
- History of malignant tumors;
- Serious liver dysfunction or chronic kidney disease (AST or ALT > 3 times the upper limit of normal, or eGFR < 30 ml/min/1.73 m2);
- Significant alcohol consumption in the past six months (Consumed more than 20 g/day for women or 30 g/day for men);
- History of serious cardiovascular or cerebrovascular disease (angina, myocardial infarction or stroke) in the past 6 months;
- History of severe gastrointestinal diseases or gastrointestinal surgery in the past 12 months;
- History of Cushing's syndrome, hypothyroidism, acromegaly, hypothalamic obesity;
- Being a smoker or having been a smoker in the 3 months prior to their screening visit;
- Taking medications affecting weight or energy intake/energy expenditure in the last 6 months, including weight loss medications, antipsychotic drugs or other medications as determined by the study physician;
- Currently participating in weight loss programs or weight change in the past 3 months (> 5% current body weight);
- Women who are pregnant or plan to become pregnant;
- Patients who cannot be followed for 24 months (due to a health situation or migration);
- Patients who are unwilling or unable to give informed consent.

5. Randomization and masking

Randomization will be conducted by the Nanfang hospital of Southern Medical University in Guangzhou, China. The randomization scheme is generated with SAS PROC PLAN (SAS Institution Inc). Although this is an open-label trial, the research assistants who collect study outcome data will be masked to participants' intervention assignment.

6. Intervention

This trial aims to compare the effect of TRE versus DCR on the improvement of NAFLD and metabolic risk factors. After the initiation of the study, we reviewed our program and prolonged the duration of intervention for 12 months in consideration of purpose of comparisons of long-term effects of TRE versus DCR on NAFLD. Therefore, the whole duration of intervention is 12 months, which included the original designed 6 months and the next 6 months follow-up visits.

6.1 Diet protocols

All participants will be instructed to follow a diet of 1500-1800kcal/d for men and 1200-1500kcal/d for women (40-55% of energy as carbohydrate, 15-20% as protein, 20-30% as fat) (*28*). Participants assigned to the TRE group will be instructed to eat from 8:00 AM to 4:00 PM and only noncaloric beverages is permitted outside of the eating window. Participants in the DCR group will be instructed to follow a calorie restriction diet without restricting eating period. All participants will be provided with one protein shake (Nutriease, Zhejiang Nutriease Co. Ltd., China) (Table 1) per day for the first 6 months and receive dietary counseling for the duration of the study. All participants will receive written dietary information booklets with portion advice and sample menus and have similar dietary energy restrictions in accordance with Dietary Guidelines for macronutrient intake (*28, 29*). After completing the initial 6 months intervention, participants will be instructed to maintain their diet regimens during the next 6-month follow-up visit.

Table 1. Nutrition Information of protein shake

**Items**	**per serving 28g**	**NRV%**
Energy	451 kJ	5%
Protein	14.4 g	24%
Fat	1.9 g	3%
Trans fat	0 g	
Carbohydrate	7.2 g	2%
Dietary Fiber	1.6 g	6%
Sodium	203 mg	10%
Vitamin A	224 μg RE	28%
Vitamin D	0.3 μg	6%
Vitamin E	3.36 mg α-TE	24%
Vitamin B1	0.39 mg	28%
Vitamin B2	0.39 mg	28%
Vitamin B6	0.39 mg	28%
Vitamin B12	0.70 μg	29%
Vitamin C	39.2 mg	39%
Nicotinic acid	3.92 mg	28%
Folic acid	112 μg DFE	28%
Pantothenic acid	1.26 mg	25%
Magnesium	36 mg	12%
Calcium	84 mg	11%
Iron	3.4 mg	23%
Zinc	2.80 mg	19%

6.2 Intervention monitoring

Participants will be encouraged to weigh foods to ensure accuracy of intake. All participants will be required to write their dietary log and record daily food picture and mealtime on a custom mobile study application (App), which will be reviewed daily by study nutritionist. Caloric intake assessment will be conducted by the nutritionist based on each participant's log and daily food photos based on the nutrient content listed in the China Food Composition Table (*30*). Intervention participants will receive follow-up telephone calls or App message twice per week, and meet with the dietician or nutritionist individually every 2 weeks to assess their adherence to the program and provide suggestions for improvements and personalized energy targets for weight maintenance in the initial 6 months intervention (Table 2).

After completing the initial 6 months intervention, participants will be instructed to maintain their diet regimens during the next 6-month follow-up visit, and write their dietary log and record food picture and mealtime three times per week. In this phase, participants received follow-up telephone calls or an App message once per week and met with the nutritionist monthly.

Table 2. Frequency of Intervention monitoring

	Months 1 - 3	Months 4 - 6	Months 7 - 12
24-hour dietary log	Everyday	Everyday	Twice to three times per week
Telephone or app message delivered guidance	Twice per week	Twice per week	Once per week
Face to face counselling	Twice to three times per month	Twice per month	Once per month

6.3 Support and education

All participants will be instructed to attend health education sessions monthly. Education sessions content will include weight measurement, dietary recall, dietary instruction (e.g., recipes, sample menus, food list, knowledge about macronutrients counting, nutrition labels reading), behavioral strategies, general health knowledge of NAFLD and metabolic disease (Table 3). All participants will be instructed not to change their physical activity habits throughout the trial.

Table 3. Topics of health education sessions

	Topics
Behavioral strategies	Introduction of exercise knowledge, guidance of exercise methods, improvement of sleep, etc.
Dietary instruction	Portions of food, knowledge about macronutrients counting, nutrition labels reading, etc.
General health knowledge	Causes and harms of NAFLD, etc.

7. Study outcomes

7.1 Primary outcome

Changes in the IHTG content from baseline at 6-month and 12-month assessment.

7.2 Secondary outcomes

1) Change in liver stiffness assessed by transient elastography (Fibroscan)
2) Change in body weight, waist circumference, and body fat composition
3) Change in cardiovascular risk factors (Blood pressure, lipids, glucose)
4) Change in insulin resistance and β cell function
5) Change in liver enzymes
6) Change in pulse wave velocity (PWV)
7) Change in Depression, quality of sleep, quality of life

8. Participant Termination Criteria

1） Occurrence of serious security issue; serious, unexpected adverse events that are directly related to the intervention.
2） Pregnancy;
3） Subjects ask to withdraw the informed consent form;
4） Unwillingness to compliant to the intervention or other unexpected reasons.

9. Data collection

9.1 Questionnaire

Medical history, personal information (age, gender, education, household income, etc.), lifestyle risk factors (cigarette smoking, alcohol drinking), physical activity, three 24-hour dietary recall, sleep quality, and quality of life.

9.2 Anthropometric measurements:

Height, weight, waist circumference, blood pressure, heart rate.

9.3 Biochemical measurements:

Overnight blood samples and spot urine will be collected to measure metabolic risk factors (fasting plasma glucose, insulin, glycosylated hemoglobin (HbA1c), serum triglycerides, total cholesterol, LDL- and HDL-cholesterol, liver enzymes, serum creatinine and urine acid. Oral glucose tolerance test (OGTT) measurement of blood glucose and serum insulin (0’, 30’,120’ min).

9.4 Transient elastography:

Transient elastography (FibroScan 502 Touch, Echosens) will be used to measure liver stiffness.

9.5 Body composition:

Whole body compositions will be quantified using a whole-body dual x-ray system (Lunar iDXA, GE Healthcare).

9.6 Body fat rate:

The bioelectrical impedance method will be used to measure body fat rate using the human body fat analyzer (V.Body HBF-371, Omron).

9.7 Visceral and subcutaneous fat area

Visceral and subcutaneous fat area will be measured using abdominal CT (Revolution, GE Healthcare) at the level of the lumbar vertebra(*31*).

9.8 The IHTG content:

The IHTG content will be measured using magnetic resonance imaging (Ingenia 3.0T mDIXON Quant, Philips Healthcare). Regions of interest (ROIs) will be selected in in the left liver lobe, the middle of the right liver lobe, and the posterior portion of the right liver lobe in the transverse sections through the right hepatic portal vein and below the second hepatic portal vein(*32-34*). All the selected ROIs should include hepatic parenchyma only, and exclude any biliary, vascular, or extrahepatic structures. The IHTG content is calculated as the mean of values from five ROIs.

10. Follow-up visits

The time points for baseline and outcome measurements are shown in Table 4.

Table 4. Follow-up Plan

	M0	M1	M2	M3	M4	M5	M6	M12
**Informed consent**	X							
**Randomization**	X							
**Demographic information**	X							
**Medical history and medications**	X			X			X	X
**Lifestyle questionnaire**	X			X			X	X
**24-hour dietary recalls *3 days**	X						X	X
**Physical and biochemical examinations**								
Weight, waist circumference	X	X	X	X	X	X	X	X
Blood pressure, heart rate	X	X	X	X	X	X	X	X
Blood glucose, HbA1c	X			X			X	X
Lipids	X			X			X	X
OGTT + insulin (0’, 30’,120’ min)	X						X	X
Liver enzymes,<br>Urine acid and creatinine	X			X			X	X
**Examinations**								
Liver MRI	X						X	X
Transient elastography	X						X	X
Abdominal CT	X						X	X

DEXA	X						X	X
PWV	X						X	X
Body fat rate (BI)	X	X	X	X	X	X	X	X
**Blood, urine and feces sample**	X						X	X
**Adverse event**	X	X	X	X	X	X	X	X
**Protein Shake**		X	X	X	X	X	X	X
**Diet counseling and health education**		X	X	X	X	X	X	X

11. Safety and adverse events

11.1 Definition of adverse events

Adverse events (AE) are defined as the occurrence of new diagnosis or deterioration of diseases during the treatment. The causal relationship to the intervention will be further evaluated.

11.2 Definition of serious adverse events

Severe adverse events (SAE) are defined as the occurrence of any acute life-threatening event, a hospitalization for any cause other than routine delivery, prolonged or permanent disability.

11.3 Surveillance and recording

Protection of participants from risks related to the intervention is of paramount concern to the physicians. All participants will be evaluated for their medical history before enrollment. Contact information of the physicians are available for all the participants.

All participants should be contacted regularly to evaluate the occurrence, severity, and duration of physical discomfort, including dizziness, fatigue, abdominal pain, headache, appetite change, constipation, or other adverse events during intervention. Evidence of the occurrence of adverse events should be based on participants' self-report rather than suggestive questioning. Any medical adverse events will be recorded and the nature, severity, and cause-effect relationship will be evaluated by physicians and recorded in a timely manner on case report forms.

11.4 Assessment of severity

The severity of adverse events is assessed as follows:

Mild: usually temporary, and not affecting daily activities;

Moderate: causing discomfort and affecting normal activities, thought tolerable and not requiring participants to take any medication.

Severe: disrupting daily activities and intolerable, requiring participants to take medication immediately.

With regard to the causal-effect relationship, medical adverse events are divided into five categories: definite, probable, possible, unlikely, and unrelated. Only "definite" and "probable" are counted as adverse events.

11.5 Treatment

Any adverse events, appropriate care or medical treatment (if necessary) should be immediately provided to keep the safety of all participants. In addition, all adverse events and serious adverse events and treatment should be recorded and reported to the Principal Investigator and the Committee within 24 hours and complete the case report form.

12. Data management

12.1. Case report form

All case report forms for each participant should be filled out by study staff in a timely manner. The case report form should be double-checked for potential errors or missing data prior to patients leaving the clinic. All data, including screening assessments, questionnaires, physical examinations, and laboratory examinations, will be filed in the participant's chart. Original documents, participants' charts, and CRF forms will be stored in the study office.

12.2. Data entry

All data will be double-entered by researcher staff. Two sets of databases will be generated and tested for consistency using the SAS program. Whenever inconsistencies are found, the data will be corrected by re-examination of the original case report forms or laboratory reports.

12.3 Data reports

Several standardized reports will be generated as follows: 1) participant recruitment and follow-up; 2) demographics; 3) data quality and monitoring; 4) adverse events. These reports will be used for study management. These reports will be blinded to research personnel who collect study outcomes.

13. Data analysis methods

Data will be analyzed according to participants' randomization assignments, regardless of their subsequent status (intention-to-treat). PROC MIXED of SAS statistical software, version 9.4 (SAS Institute Inc), will be used to obtain point estimates and SEs of the treatment effects and to test for differences between treatments. Group differences in the study outcomes will be evaluated using the general linear model for continuous variables and the chi-squared test for categorical variables. A mixed-effects model will be used to assess the effects of diet programs on the change in IHTG content and main outcomes and an autoregressive correlation matrix will be used to correct within-participant correlation for repeated measurements. In this model, participants will be assumed to be random effects, and intervention group, time, and their 2-factor interactions will be assumed to be estimable fixed effects. Multiple imputations for missing data in the multivariable analyses will be conducted using the Markov chain Monte Carlo method. Data will be presented as least-squares means with 95% confidence intervals (CIs). $P < 0.05$ will be considered statistically significant.

14. Training for study staff

All study staff (physicians and nurses) will be trained and certified before the study is initiated. The training program will include instruction on questionnaire administration and anthropometrics (height, weight, waist circumference, and hip girth) and blood pressure measurement. A standard questionnaire will be used, which includes personal information (name, sex, age, place of birth, marital status, occupation, education, household income, etc.), medical history, medication use, family history, lifestyle factors (smoking and drinking), physical activity, dietary habits, and menstrual and reproductive history. Anthropometric measurements and blood pressure will be obtained using a standard protocol. The data, including that for exercise intensity, duration, heart rate, and anthropometric measurements, will be collected and recorded in CRFs by nurses every week. All technologists are experienced and masked to participant randomization.

15.Quality Control

China's Standard for Quality Management of Clinical Trials (GCP) and other relevant laws and regulations on clinical trials will be complied with. The standard quality control process will be carried out at each step of the study, including repeated laboratory measurements in 5-10% of random blind samples, and all data will be entered independently.

Establish a research procedure manual (MOP): we will develop a standardized process of data collection, which will include the recruitment of subjects, instructions for the use of tables, intervention programs, index measurement, specimen collection and management, etc., to guide all tabular information entry and diagnosis and treatment processes, and other aspects of the study.

Training: all researchers will receive relevant training before the start of the study, including standardized processes, recruitment processes, follow-up, intervention programs, measurement procedures, and the use of MOP. At the same time, regular retraining sessions will be conducted to ensure the quality of research.

Quality monitoring and reporting: quality control personnel will regularly review the QC data, including the timeliness of the completion of the visit, data collection, program implementation and data quality.

16. Organization and Communication

A steering committee (Steering committee, SC), chaired by Drs Huijie Zhang and Yikai Xu, Nanfang Hospital, Southern Medical University, will be responsible for the study. The research team consists of researchers and health management team members from the Norte Health Management Center. the steering committee (SC) is responsible for organizing and supervising research implementation, approving the research program, including modification of the research program, monitoring the recruitment and withdrawal process of subjects, implementation of intervention programs, recruitment and intervention of subjects, data collection and quality control, and responding to questions raised by the ethics body review committee (Institutional review board, IRB), etc. The research center will be involved in the recruitment, intervention, data collection and quality control of the subjects. the research center will do randomization and data analysis, and help prepare to report research results and papers for publication. In order to facilitate communication between investigators and researchers, regular working meetings will be held. SC will discuss relevant scientific and management issues in the study with three live meetings a year and monthly conference calls. Among them, important information, such as recruitment progress, data completion and quality, and compliance with intervention programs, will be sent monthly by e-mail to committee members and key staff.

17. Ethics Approval

This clinical trial must comply with the Helsinki Declaration (1996 edition) and the relevant regulations of Chinese clinical trials. Before the trial begins, the hospital ethics committee of the research unit shall review the trial plan and issue the approval document before the implementation of the trial plan.

For all research program amendments (excluding administrative amendments and amendments that have no impact on the implementation of subjects, data or trials), the amendment and the applicable informed consent must be submitted to the Ethics Committee for approval immediately prior to the implementation of these changes. The researcher is responsible for ensuring that the conditions approved by the study are met and that the trial correction program or serious adverse events are reported to the ethics committee or the corresponding organization as required by the ethics committee.

All subjects will be given the opportunity to read the entire informed consent document and, by the researcher or authorized researcher, to provide the subjects, in writing, with a complete and comprehensive presentation of the background, purpose, research methods, research process, test items to be involved, individual rights and obligations, possible risks, and possible benefits of the research process. The subjects had enough time to ask questions, and the researchers needed to provide truthful and accurate answers in plain language. The subjects had sufficient time to discuss with other family members whether to participate in the study. The subjects should know that they can withdraw from the study at any time without any reason; the subjects agree to collect and apply the data related to the study and are willing to cooperate with the follow-up. When they exit, the subjects are asked whether they agree to apply the collected data. Participants will be required to sign informed consent before they can be selected for clinical trials. Informed consent shall be kept for reference as one of the original materials for clinical trials.

18. Proposed study timeline

The clinical study is expected to take about 3 years after registration in the clinicaltrials.gov (Identifier: NCT03786523 and NCT04988230), the trial is expected to take about 3 years to carry out this clinical study, in which MOP development and personnel training and subject recruitment time is 1 year. Intervention and data collection cycle is 18 months, data entry and analysis and paper writing lasted about 0.5 years.

References

1. J. D. Browning *et al.*, Prevalence of hepatic steatosis in an urban population in the United States: impact of ethnicity. *Hepatology* **40**, 1387-1395 (2004).
2. C. D. Williams *et al.*, Prevalence of nonalcoholic fatty liver disease and nonalcoholic steatohepatitis among a largely middle-aged population utilizing ultrasound and liver biopsy: a prospective study. *Gastroenterology* **140**, 124-131 (2011).
3. C. Garcia-Monzon *et al.*, Characterization of pathogenic and prognostic factors of nonalcoholic steatohepatitis associated with obesity. *Journal of hepatology* **33**, 716-724 (2000).
4. P. Gupte *et al.*, Non-alcoholic steatohepatitis in type 2 diabetes mellitus. *Journal of gastroenterology and hepatology* **19**, 854-858 (2004).
5. G. Musso, R. Gambino, M. Cassader, G. Pagano, Meta-analysis: natural history of non-alcoholic fatty liver disease (NAFLD) and diagnostic accuracy of non-invasive tests for liver disease severity. *Annals of medicine* **43**, 617-649 (2011).
6. G. Targher, C. P. Day, E. Bonora, Risk of cardiovascular disease in patients with nonalcoholic fatty liver disease. *The New England journal of medicine* **363**, 1341-1350 (2010).
7. P. Haentjens *et al.*, Identifying non-alcoholic fatty liver disease among asymptomatic overweight and obese individuals by clinical and biochemical characteristics. *Acta clinica Belgica* **64**, 483-493 (2009).
8. J. C. Cohen, J. D. Horton, H. H. Hobbs, Human fatty liver disease: old questions and new insights. *Science* **332**, 1519-1523 (2011).
9. A. M. Cali *et al.*, Intrahepatic fat accumulation and alterations in lipoprotein composition in obese adolescents: a perfect proatherogenic state. *Diabetes care* **30**, 3093-3098 (2007).
10. L. Shen, Treatment implication from the potential association between nonalcoholic fatty liver disease and cardiovascular disease. *Hepatology* **54**, 377-378 (2011).
11. J. B. Schwimmer, P. E. Pardee, J. E. Lavine, A. K. Blumkin, S. Cook, Cardiovascular risk factors and the metabolic syndrome in pediatric nonalcoholic fatty liver disease. *Circulation* **118**, 277-283 (2008).
12. M. Hamaguchi *et al.*, The metabolic syndrome as a predictor of nonalcoholic fatty liver disease. *Annals of internal medicine* **143**, 722-728 (2005).
13. M. Romero-Gomez, S. Zelber-Sagi, M. Trenell, Treatment of NAFLD with diet, physical activity and exercise. *Journal of hepatology* **67**, 829-846 (2017).
14. N. Chalasani *et al.*, The diagnosis and management of nonalcoholic fatty liver disease: Practice guidance from the American Association for the Study of Liver Diseases. *Hepatology* **67**, 328-357 (2018).
15. P. Sumithran *et al.*, Long-term persistence of hormonal adaptations to weight loss. *The New England journal of medicine* **365**, 1597-1604 (2011).
16. G. Diabetes Prevention Program Research *et al.*, 10-year follow-up of diabetes incidence and weight loss in the Diabetes Prevention Program Outcomes Study. *Lancet* **374**, 1677-1686 (2009).
17. A. R. G. Look, Eight-year weight losses with an intensive lifestyle intervention: the look AHEAD study. *Obesity* **22**, 5-13 (2014).
18. K. R. Middleton, S. D. Anton, M. G. Perri, Long-Term Adherence to Health Behavior Change. *American journal of lifestyle medicine* **7**, 395-404 (2013).
19. R. V. Seimon *et al.*, Do intermittent diets provide physiological benefits over continuous diets for weight loss? A systematic review of clinical trials. *Molecular and cellular endocrinology* **418 Pt 2**, 153-172 (2015).
20. M. P. Mattson, V. D. Longo, M. Harvie, Impact of intermittent fasting on health and disease processes. *Ageing Res Rev* **39**, 46-58 (2017).
21. A. Hodge *et al.*, Non-alcoholic fatty liver disease Intermittent Fasting Time Intervention (NIFTI): Fasting without calorie

restriction improves hepatic transient elastography, visceral adiposity and insulin resistance compared to standard care. *Hepatology* **60**, 632a-632a (2014).

22. H. Kahleova *et al.*, Eating two larger meals a day (breakfast and lunch) is more effective than six smaller meals in a reduced-energy regimen for patients with type 2 diabetes: a randomised crossover study. *Diabetologia* **57**, 1552-1560 (2014).
23. E. F. Sutton *et al.*, Early Time-Restricted Feeding Improves Insulin Sensitivity, Blood Pressure, and Oxidative Stress Even without Weight Loss in Men with Prediabetes. *Cell Metab* **27**, 1212-1221 e1213 (2018).
24. S. Gill, S. Panda, A Smartphone App Reveals Erratic Diurnal Eating Patterns in Humans that Can Be Modulated for Health Benefits. *Cell Metab* **22**, 789-798 (2015).
25. G. M. Tinsley *et al.*, Time-restricted feeding in young men performing resistance training: A randomized controlled trial. *European journal of sport science* **17**, 200-207 (2017).
26. J. D. LeCheminant, E. Christenson, B. W. Bailey, L. A. Tucker, Restricting night-time eating reduces daily energy intake in healthy young men: a short-term cross-over study. *The British journal of nutrition* **110**, 2108-2113 (2013).
27. H. Kahleova *et al.*, Erratum to: Eating two larger meals a day (breakfast and lunch) is more effective than six smaller meals in a reduced-energy regimen for patients with type 2 diabetes: a randomised crossover study. *Diabetologia* **58**, 205 (2015).
28. R. H. Eckel *et al.*, 2013 AHA/ACC guideline on lifestyle management to reduce cardiovascular risk: a report of the American College of Cardiology/American Heart Association Task Force on Practice Guidelines. *J Am Coll Cardiol* **63**, 2960-2984 (2014).
29. C. N. Society, *Dietary Guidelines for Chinese Residents*. (People's Medical Publishing House, Beijing, China, ed. 1, 2016).
30. Y. Yang, *China Food Composition Tables Standard Edition*. (Beijing University Medical Press, Beijing, China, ed. 6, 2018).
31. Y. Tong, J. K. Udupa, D. A. Torigian, Optimization of abdominal fat quantification on CT imaging through use of standardized anatomic space: a novel approach. *Medical physics* **41**, 063501 (2014).
32. S. D. Serai, J. R. Dillman, A. T. Trout, Proton Density Fat Fraction Measurements at 1.5- and 3-T Hepatic MR Imaging: Same-Day Agreement among Readers and across Two Imager Manufacturers. *Radiology* **284**, 244-254 (2017).
33. Y. Zhang *et al.*, Comparison of CT and magnetic resonance mDIXON-Quant sequence in the diagnosis of mild hepatic steatosis. *The British journal of radiology* **91**, 20170587 (2018).
34. G. M. Kukuk *et al.*, Comparison between modified Dixon MRI techniques, MR spectroscopic relaxometry, and different histologic quantification methods in the assessment of hepatic steatosis. *European radiology* **25**, 2869-2879 (2015).

1.3 Summary of change of protocol

We revised the design of study (6-month intervention) and prolonged the duration of intervention to 12 months in consideration of purpose of comparisons of long-term effects of TRE versus DCR on NAFLD.

Summary of changes of protocol

**Changes**	**Original protocol**	**Final protocol**
Version	1.0	2.0
Date	2018-12-5	2021-3-16
Intervention duration	6 months	12 months
Intervention		After completing the initial 6 months intervention, participants will be instructed to maintain their diet regimens during the next 6-month follow-up visit, and write their dietary log and record food picture and mealtime three times per week. In this phase, participants received follow-up telephone calls or an App message once per week and met with the nutritionist monthly.
Primary outcome	Change in the IHTG content from baseline to 6 months	Change in the IHTG content from baseline to 6 months and 12 months

Statistical Analysis Plan

Statistical power and detectable effect sizes

This study is a randomized, open label, parallel controlled study. Randomization will be conducted at the research center, and random coding will be generated by SAS in SAS PROC PLAN. The research assistants who collect study outcome data will be masked to participants' intervention assignment. Subjects will be randomly assigned to TRE group or DCR group.

The sample size of this study is calculated according to the primary outcome. In this study, the primary outcome is the change of intrahepatic triglyceride (IHTG) content in obese patients. This trial is designed to provide more than 90% statistical power to detect a significant difference of 0.8% in IHTG content (SD, 1.0%) between the TRE group and the DCR group at a significance level of 0.05 using a 2-tailed test. The proposed group difference and SD of reduction in IHTG content are based on data from previous study(*22*). We also assumed an 80% follow-up rate. We will recruit 86 participants, 43 in each group for the study.

Data analysis plan

Continuous variables that are normally distributed will be represented as Mean ± standard deviation (SD). Data that are not normally distributed will be presented as median (quartile interval). Categorical variables will be presented as frequency (percentage).

Data will be analyzed according to participants' randomization assignment (intent-to treat). PROC MIXED of SAS statistical software, version 9.4 (SAS Institute Inc), will be used to obtain point estimates and SEs of the treatment effects and to test for differences between treatments. The study end-point indicators include categorical and continuous variables. The categorical variables will be analyzed by chi-squared test, logistic regression, and GEE model. The analyses of the single continuous outcomes will use a mixed effects model implemented using PROC MIXED or PROC GLIMMIX of SAS version 9.4. In this model, participants will be assumed to be random effects, and intervention group, time, and their 2-factor interactions will be assumed to be estimable fixed effects. An autoregressive correlation matrix will be used to correct within-participant correlation for repeated measurements. Multiple imputations for missing data in the multivariable analyses will be conducted using the Markov chain Monte Carlo method. Data will be presented as least-squares means with 95% confidence intervals (CIs). $P < 0.05$ is considered statistically significant.

1.Descriptive statistical analysis of baseline data

The baseline data include demographic information, baseline dietary calorie intake and micronutrition, alcohol intake, physical activity, body weight, BMI, blood pressure, blood glucose, HOMA-IR, lipids, body fat percent, body lean mass, abdominal total fat area, subcutaneous fat area, the IHTG content, and liver stiffness measurement. The continuous variables distributed normally will be represented as Mean ± SD, the categorical variables will be presented as frequency (percentage), and the continuous variables that are not normally distributed will be presented as the median (quartile interval).

2.Statistical analysis of outcomes

Primary outcome

The primary outcome is the change of the IHTG content at Month 6 and Month 12 compared to the baseline. The primary outcome data will be analyzed by PROC MIXED or PROC GLIMMIX model of SAS 9.4. A mixed-effects model will be used to assess the effects of diet interventions on the change of IHTG content and an autoregressive correlation matrix will be used to correct within-participant correlation for repeated measurements. In this model, participant is assumed to be random effects, and group, time, and their 2-factor interactions are assumed to be estimable fixed effects. Multiple imputations for missing data in the multivariable analyses will be conducted using the Markov chain Monte Carlo method.

Secondary outcomes

The secondary study outcomes include changes of body weight, BMI, waist circumference, body fat, abdominal fat area, abdominal subcutaneous fat area, and lean mass from baseline to 6 months. The changes in cardiovascular risk factors and liver enzymes at 6 months and 12 months from baseline will be assessed. Cardiovascular risk factors include blood pressure, lipids, fasting glucose and HOMA-IR. Liver enzymes include alanine aminotransferase, aspartate aminotransferase and γ-glutamyl transpeptidase. $P < 0.05$

will be considered statistically significant. A mixed effects model will be used for the analyses of continuous variables. Chi-squared test and logistic regression analysis and GEE model will be used for the categorical variables. An autoregressive correlation matrix will be used to correct within-participant correlation for repeated measurements. Multiple imputations for missing data in the multivariable analyses will be conducted using the Markov chain Monte Carlo method.

3. Adverse events

The incidence of adverse events (AE) between the two groups will be analyzed by Chi-squared test and logistic regression analysis and GEE model.
