# Supplementary material for: Effects of Time-Restricted Eating on Nonalcoholic Fatty Liver Disease: The TREATY-FLD Randomized Clinical Trial
Source: JAMA Netw Open. 2023 Mar 17;6(3):e233513. doi: 10.1001/jamanetworkopen.2023.3513 (PMC10024204; doi:10.1001/jamanetworkopen.2023.3513)
Supplement: Supplement 2. — eTable 1. Diet Adherence and Physical Activity During Intervention eTable 2. Sensitivity Analyses of the Main Outcomes eTable 3. Adverse Events During Intervention eFigure 1. Effect of Diets on the Intrahepatic Triglyceride (IHTG) Content by Subgroups of Adherence eFigure 2. Effect of Time-Restricted Eating Versus Daily Calorie Restriction on the Weight and Waist Circumference [file jamanetwopen-e233513-s002.pdf]

Supplementary Online Content

Wei X, Lin B, Huang Y, et al. Effects of time-restricted eating on nonalcoholic fatty liver disease: the TREATY-FLD randomized clinical trial. *JAMA Netw Open*. 2023;6(3):e233513. doi:10.1001/jamanetworkopen.2023.3513

eTable 1. Diet Adherence and Physical Activity During Intervention

eTable 2. Sensitivity Analyses of the Main Outcomes

eTable 3. Adverse Events During Intervention

eFigure 1. Effect of Diets on the Intrahepatic Triglyceride (IHTG) Content by Subgroups of Adherence

eFigure 2. Effect of Time-Restricted Eating Versus Daily Calorie Restriction on the Weight and Waist Circumference

This supplementary material has been provided by the authors to give readers additional information about their work.

© 2023 Wei X et al. *JAMA Network Open.*

eTable 1. Diet Adherence and Physical Activity during Intervention.

Variables	TRE (n = 45)	DCR (n = 43)	*P* value
Diet compliance,mean (SD)，% [a]			
Month 6	87.9 ± 10.3	90.5 ± 11.7	.29
Month 12	85.0 ± 10.7	85.7 ± 9.4	.77
Energy deficit, mean (95% CI), kcal/d			
Month 6	788.5 (717.5 to 859.4)	718.4 (648.4 to 788.4)	.17
Month 12	684.7 (608.1 to 761.3)	586.9 (516.0 to 657.9)	.07
Carbohydrate intake, mean (SD), %			
Month 6	51.3 ± 3.6	51.7 ± 3.6	.62
Month 12	51.2 ± 3.4	51.1 ± 3.3	.96
Protein intake,mean (SD)，%			
Month 6	19.7 ± 1.3	19.2 ± 1.4	.07
Month 12	19.3 ± 1.0	19.0 ± 1.3	.27
Fat intake, mean (SD) ，%			
Month 6	29.3 ± 3.2	29.3 ± 3.0	.97
Month 12	29.9 ± 3.1	30.2 ± 2.7	.74
Start of eating window, mean (SD), local time hour: minute			
Month 6	8:36 ± 00:55	8:27 ± 1:08	.56
Month 12	8:15 ± 00:53	8:40 ± 1:11	.10
End of eating window, mean (SD), local time hour: minute			
Month 6	16:20 ± 0:17	19:19 ± 0:29	<.001
Month 12	16:36 ± 0:23	19:32 ± 0:26	<.001
Daily eating window, mean (SD), hour: minute			
Month 6	7:44 ± 0:53	10:52 ± 1:12	<.001
Month 12	8:20 ± 0:51	10:52 ± 1:11	<.001
Physical activity, median (IQR), METs/week			
Month 6	10.0 (3.1 - 17.6)	9.9 (6.6 - 16.6)	.53
Month 12	14.8 (9.9 - 26.4)	11.0 (6.1 - 19.9)	.07
SF-12, mean (SD)			
Physical component summary			
Month 6	47.4 (8.2)	47.9 (7.0)	.77
Month 12	48.0 (8.0)	47.8 (6.7)	.92
Mental component summary			
Month 6	53.9 (7.7)	54.1 (6.8)	.93
Month 12	52.5 (7.9)	52.9 (7.5)	.81
Total PSQI sleep quality score, mean (SD)			
Month 6	4.9 (2.3)	5.1 (1.8)	.61
Month 12	5.3 (2.4)	5.7 (2.9)	.54

© 2023 Wei X et al. *JAMA Network Open.*

Total PHQ-9 score, median (IQR)			
Month 6	2 (0 - 4)	3 (1 - 4)	.84
Month 12	2 (0 - 5)	2.5 (1 – 5.5)	.51

Abbreviation: TRE, time-restricted eating; DCR, daily calorie restriction; METs, metabolic equivalents; SF-12, the 12-item Short-Form Health Survey Questionnaire; PSQI, the Pittsburgh Sleep Quality Index; PHQ, Patient Health Questionnaire.

[a] Compliance to the diets was defined as the percentage of adherent days during the 6-month and 12-month intervention

© 2023 Wei X et al. *JAMA Network Open.*

eTable 2. Sensitivity Analyses of the Main Outcomes [a].

	Changes (95% CIs)		Difference between groups (95% CIs)	*P* Value
	TRE (n = 45)	DCR (n = 43)		
**Primary outcome**				
Intrahepatic triglyceride content, %				
Month 6	-8.2 (-10.5 to -6.0)	-7.8 (-10.0 to -5.6)	-0.4 (-3.7 to 2.8)	.80
Month 12	-6.9 (-9.4 to -4.3)	-7.5 (-10.0 to -5.1)	0.7 (-2.8 to 4.1)	.70
Intrahepatic triglyceride content, % [b]				
Month 6	-7.5 (-9.5 to -5.5)	-7.4 (-9.3 to -5.4)	-0.1 (-2.9 to 2.6)	.92
Month 12	-7.4 (-9.6 to -5.3)	-8.2 (-10.3 to -6.0)	0.7 (-2.1 to 3.6)	.61
**Secondary outcome**				
Weight, kg				
Month 6	-9.9 (-11.9 to -7.8)	-9.5 (-11.6 to -7.4)	-0.4 (-3.4 to 2.5)	.79
Month 12	-7.6 (-10.1 to -5.2)	-7.6 (-9.8 to -5.4)	-0.0 (-3.3 to 3.2)	>.99
BMI, kg/cm^2				
Month 6	-3.6 (-4.4 to -2.9)	-3.4 (-4.1 to -2.6)	-0.3 (-1.3 to 0.8)	.63
Month 12	-2.8 (-3.7 to -2.0)	-2.7 (-3.5 to -1.9)	-0.1 (-1.3 to 1.0)	.86
Waist circumference, cm				
Month 6	-9.9 (-12.2 to -7.7)	-8.9 (-11.2 to -6.7)	-1.0 (-4.2 to 2.2)	.55
Month 12	-8.6 (-11.2 to -5.9)	-8.0 (-10.3 to -5.6)	-0.6 (-4.1 to 2.9)	.73
Fat mass, kg				
Month 6	-7.2(-8.8 to -5.6)	-6.9 (-8.4 to -5.3)	-0.3 (-2.6 to 1.9)	.77
Month 12	-5.8 (-7.7 to -3.8)	-5.4 (-7.1 to -3.7)	-0.3 (-2.8 to 2.2)	.78
Visceral fat, cm^2				
Month 6	-39.9 (-51.9 to -27.9)	-37.7 (-49.5 to -26.0)	-2.2 (-19.2 to 14.8)	.80
Month 12	-32.6 (-46.6 to -18.6)	-34.8 (-46.8 to -22.8)	2.2 (-16.4 to 20.8)	.82

Abbreviation: TRE, time-restricted eating; DCR, daily calorie restriction; BMI, body mass index (calculated as the weight in kilograms divided by height in meters squared).

[a] Results are from sensitivity analyses using multiple imputation techniques to impute missing values.

[b] Adjusted for weight loss.

© 2023 Wei X et al. *JAMA Network Open.*

eTable 3. Adverse Events during Intervention.

Adverse events	TRE (n = 45)	DCR (n = 43)	*P* value
Discomfort in stomach, n (%)	2 (4%)	2 (5%)	.98
Constipation, n (%)	2 (4%)	0	.16
Dyspepsia, n (%)	5 (11%)	4 (9%)	.75
Hunger, n (%)	6 (13%)	3 (7%)	.31
Decreased appetite, n (%)	0	2 (5%)	.15
Dizziness, n (%)	5 (11%)	4 (9%)	.75
Fatigue, n (%)	2 (4%)	2 (5%)	.98
Total cases, n (%)	13 (29%)	12 (28%)	.87

Abbreviation: TRE, time-restricted eating; DCR, daily calorie restriction.

Advent events from randomization among all participants are included in the table and are presented with the numbers of participants that reported adverse events. In addition, the total is the number of participants with any adverse events.

© 2023 Wei X et al. *JAMA Network Open.*

eFigure 1. Effect of diets on the Intrahepatic Triglyceride (IHTG) Content by Subgroups of Adherence.

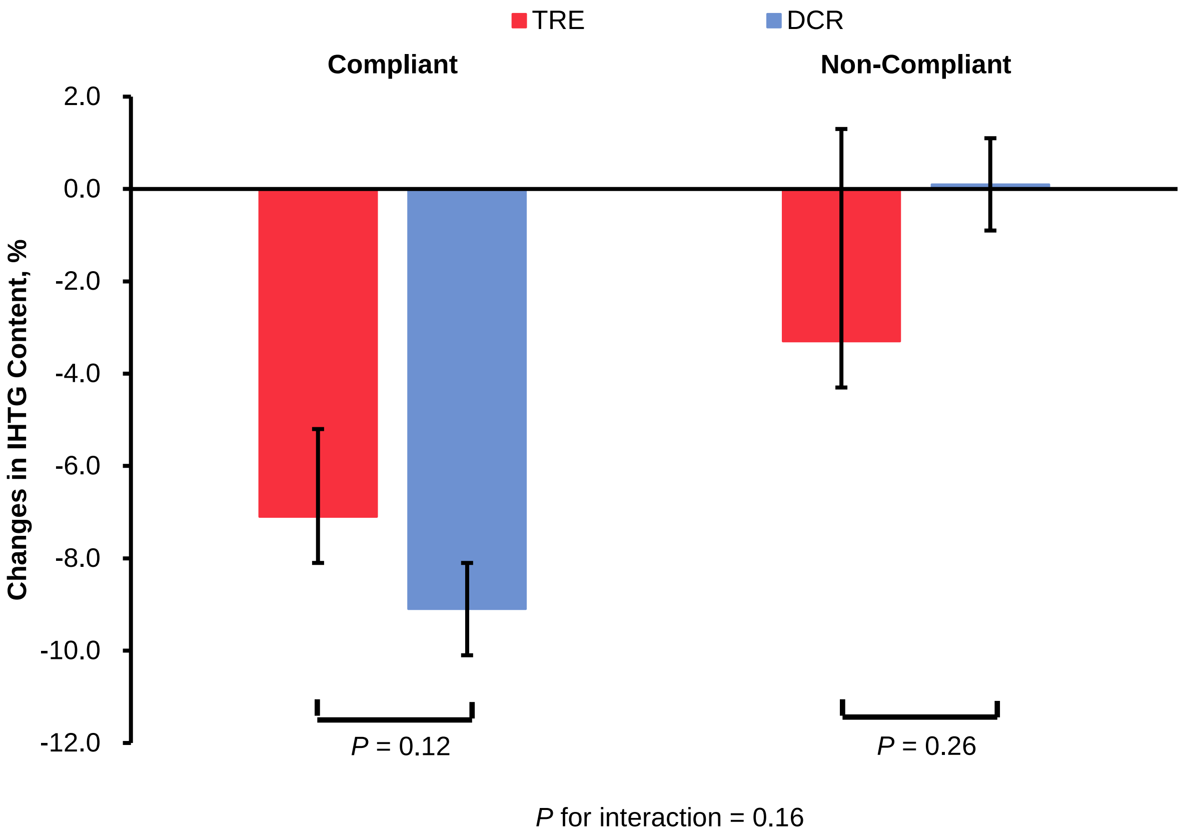

Compliant is defined as adhering to the diet program more than 75% of the days. Non-Compliant is defined as adhering to the diet program less than 75% of the days.

Data are presented as estimated absolute IHTG content at measurements times, by treatment group, from linear mixed models. Error bars represent 95% Cis.

Abbreviation: TRE, time-restricted eating; DCR, daily calorie restriction.

© 2023 Wei X et al. *JAMA Network Open.*

eFigure 2. Effect of Time-Restricted Eating versus Daily Calorie Restriction on the Weight and Waist Circumference.

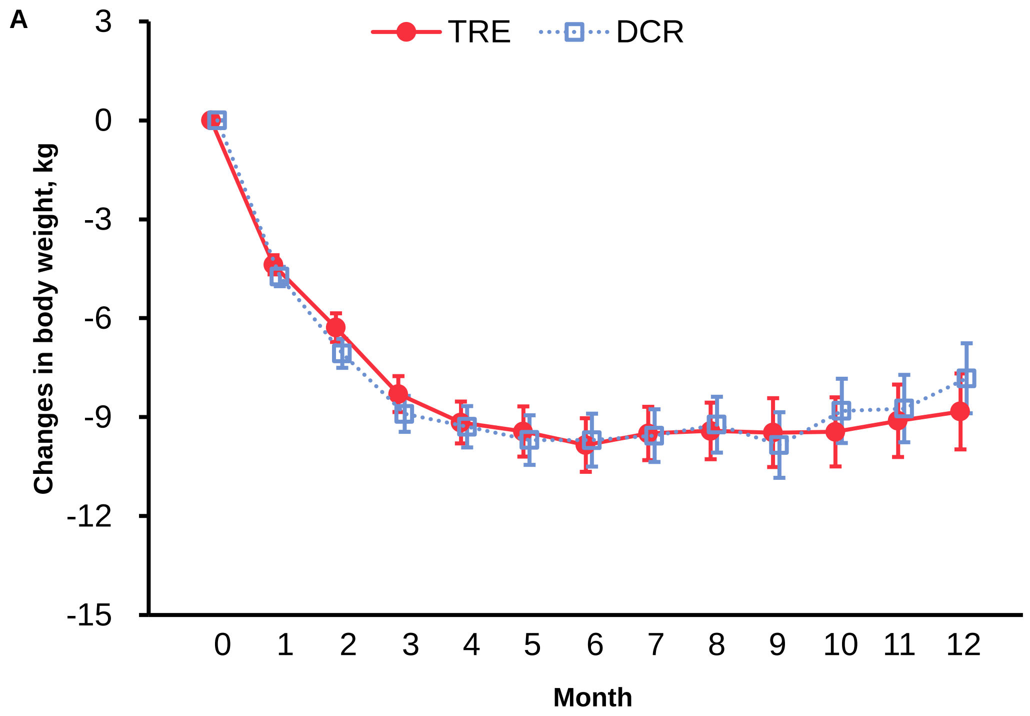

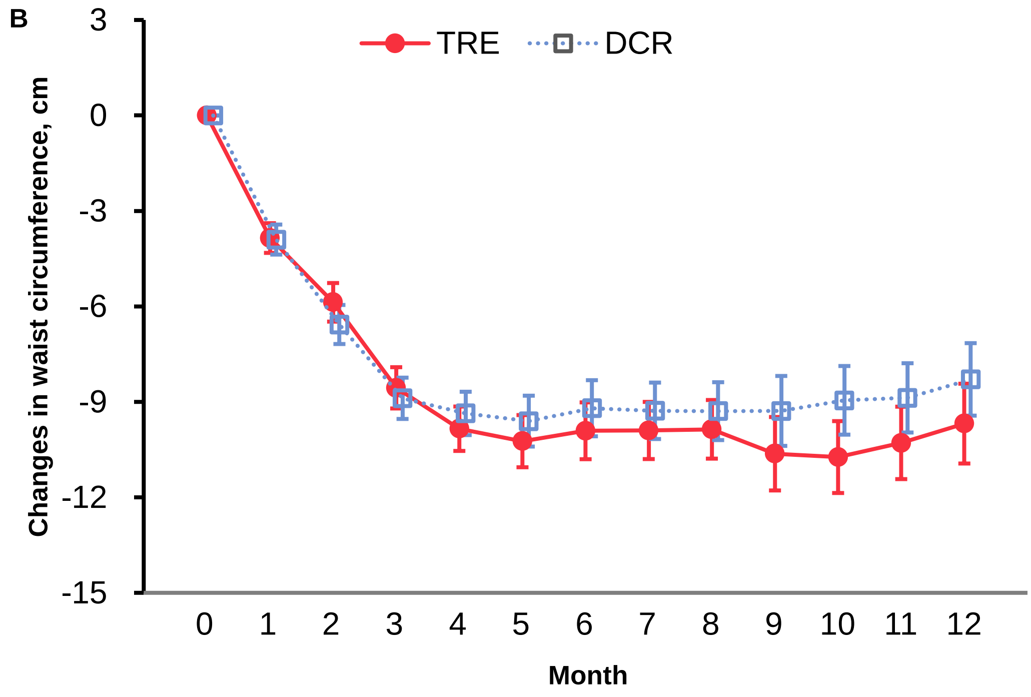

Data are presented as observed values at measurements times, by treatment group. Error bars represent 95% CIs.

Panel A. Change in body weight over time for TRE group (red line) and DCR group (blue line).

Panel B. Change in waist circumference over time for TRE group (red line) and DCR group (blue line).

Abbreviation: TRE, time-restricted eating; DCR, daily calorie restriction.

© 2023 Wei X et al. *JAMA Network Open.*
