# Supplementary material for: Effects of Time-Restricted Eating on Nonalcoholic Fatty Liver Disease: The TREATY-FLD Randomized Clinical Trial
Source: JAMA Netw Open. 2023 Mar 17;6(3):e233513. doi: 10.1001/jamanetworkopen.2023.3513 (PMC10024204; doi:10.1001/jamanetworkopen.2023.3513)
Supplement: Supplement 3. — Data Sharing Statement [file jamanetwopen-e233513-s003.pdf]

Data Sharing Statement

Wei. Effects of Time-Restricted Eating on Nonalcoholic Fatty Liver Disease. *JAMA Netw Open*. Published March 17, 2023. doi:10.1001/jamanetworkopen.2023.3513

Data
Data available: No
